# Gut microbiome dysregulation drives bone damage in broiler tibial dyschondroplasia by disrupting glucose homeostasis

**DOI:** 10.1038/s41522-022-00360-6

**Published:** 2023-01-03

**Authors:** Ting-ting Xu, Pan Chen, Chao-dong Zhang, Aftab Shaukat, Lu-xi Lin, Ke Yue, Wen-li Ding, Xishuai Tong, Kai-li Liu, Yan-feng He, Jing-fei Xie, Fang Liu, Cai Zhang, Huai-yong Zhang, Shu-cheng Huang

**Affiliations:** 1 College of Veterinary Medicine, Henan Agricultural University, Zhengzhou, 450002 China; 2 National Center for International Research on Animal Genetics, Breeding and Reproduction (NCIRAGBR), Huazhong Agricultural University, Wuhan, 430070 China; 3 Institutes of Agricultural Science and Technology Development (Joint International Research Laboratory of Agriculture and Agri-Product Safety of the Ministry of Education of China), College of Veterinary Medicine, Yangzhou University, Yangzhou, 225009 China; 4 Laboratory of Environment and Livestock Products, Henan University of Science and Technology, Luoyang, 471023 China; 5 College of Animal Science and Technology, Key Laboratory of Animal Biochemistry and Nutrition, Ministry of Agriculture, Henan Agricultural University, Zhengzhou, 450046 China

**Keywords:** Applied microbiology, Microbial communities

## Abstract

Tibial dyschondroplasia (TD) with multiple incentives is a metabolic skeletal disease that occurs in fast-growing broilers. Perturbations in the gut microbiota (GM) have been shown to affect bone homoeostasis, but the mechanisms by which GM modulates bone metabolism in TD broilers remain unknown. Here, using a broiler model of TD, we noted elevated blood glucose (GLU) levels in TD broilers, accompanied by alterations in the pancreatic structure and secretory function and damaged intestinal barrier function. Importantly, faecal microbiota transplantation (FMT) of gut microbes from normal donors rehabilitated the GM and decreased the elevated GLU levels in TD broilers. A high GLU level is a predisposing factor to bone disease, suggesting that GM dysbiosis-mediated hyperglycaemia might be involved in bone regulation. 16S rRNA gene sequencing and short-chain fatty acid analysis revealed that the significantly increased level of the metabolite butyric acid derived from the genera *Blautia* and *Coprococcus* regulated GLU levels in TD broilers by binding to GPR109A in the pancreas. Tibial studies showed reduced expression of vascular regulatory factors (including PI3K, AKT and VEFGA) based on transcriptomics analysis and reduced vascular distribution, contributing to nonvascularization of cartilage in the proximal tibial growth plate of TD broilers with elevated GLU levels. Additionally, treatment with the total flavonoids from *Rhizoma drynariae* further validated the improvement in bone homoeostasis in TD broilers by regulating GLU levels through the regulation of GM to subsequently improve intestinal and pancreatic function. These findings clarify the critical role of GM-mediated changes in GLU levels via the gut–pancreas axis in bone homoeostasis in TD chickens.

## Introduction

Tibial dyschondroplasia (TD) is a common problem occurring in broiler legs. The current prevalence of intensive farming has prompted the poultry industry to focus excessively on the growth rate of broilers, which has resulted in bones that cannot cope with the increased weight, ultimately increasing the incidence of TD in broilers^1,2^. The clinical symptoms of TD broiler chickens include slow movement, difficulty standing, walking with split legs or wings, decreased growth performance, and even death^1,3,4^. According to statistics, TD accounts for 30% of the incident bone disease in broilers worldwide, while the incidence of TD is approximately 10% in China^2,5^. In addition, skeletal growth and development problems caused by TD in broilers have resulted in economic losses of up to $150 million in the United States^6^. More seriously, poultry skeletal growth and development problems also lead to a reduction in carcass quality^7,8^. This decrease in quality might lead to a further decrease in indirect reprocessing profits and reduce the gross profits of the poultry industry (by approximately 10–40%)^7^. TD has attracted more attention to poor animal welfare and causing huge economic losses to the flourishing poultry industry. Several studies have shown that some factors, including vasculature dysfunction^1^, high blood glucose levels^9^ and variations in the gut microbiota (GM)^10^, substantially affect TD occurrence in broilers. However, the causes and specific pathogenesis of TD in chickens are still unknown.

Numerous studies indicate that high blood glucose levels impair bone formation, inhibit bone mineralisation and affect osteoblast differentiation^11–13^. A continuous increase in blood glucose levels reduces nitric oxide (NO) activity, affects vasodilatory function, stimulates blood vessels to produce a large number of cytokines, promotes the inflammatory response and oxidative stress, improves vascular permeability, and causes vascular endothelial damage^14^. Research experiments by Rath et al.^15^ and Huang et al.^1^ revealed that the distribution of blood vessels in the tibial growth plate (TGP) region is significantly reduced upon inhibiting the expression of vascular endothelial growth factor (VEGF), causing TD lesions. Furthermore, a substantial reduction in blood vessels distributed in the tibia reduces the required supply of oxygen, and a serious deficiency in nutrients required for chondrocyte growth has been noted. These changes lead to chondrocyte differentiation and maturation and the production of an abnormal matrix, which postpones the process of bone calcification and remodelling and accelerates TD occurrence and development^3,16^. Moreover, our previous study revealed elevated blood glucose levels in TD broilers^9^. Therefore, we speculate that high glucose levels may cause TD lesions by damaging blood vessels in broilers.

The gut–pancreas axis plays a key role in regulating glucose homoeostasis and may be involved in the treatment of type 2 diabetes, hypoglycaemia and hyperinsulinaemia^17–19^. When stimulated by nutrition, the gut secretes a number of peptide hormones to increase insulin release, while insulin secretion is also regulated by the G protein-coupled receptor (GPCR)^17,20^. These mechanisms lower blood glucose levels by stimulating cellular glucose uptake. In addition, GM dysbiosis affects insulin signalling and glucose metabolism by influencing metabolic toxicity, the inflammatory response and insulin intolerance^21^. Increased plasma glucose levels are associated with significant decreases in Bacteroidetes and increased relative levels of Firmicutes^22^. Cani et al.^23^ found that improved GM dysbiosis mitigates insulin resistance in diabetic mice. In addition, the dysregulated abundance and diversity of the GM have also been noted in the pathogenesis of TD broilers^9,24^. Furthermore, the GM is a critical regulator of bone, and alterations in its composition contribute to pathological bone loss or a lower bone density^25^. The GM may be involved in the development of TD through changes in glucose levels mediated by the gut–pancreas axis. However, the mechanism of action remains unclear. Therefore, the present study aimed to investigate the mechanism by which GM dysbiosis regulates TD through gut–pancreas axis-mediated glucose homoeostasis.

## Results

### Elevated blood glucose levels and damaged pancreas in TD broilers

**Fig. 1:**
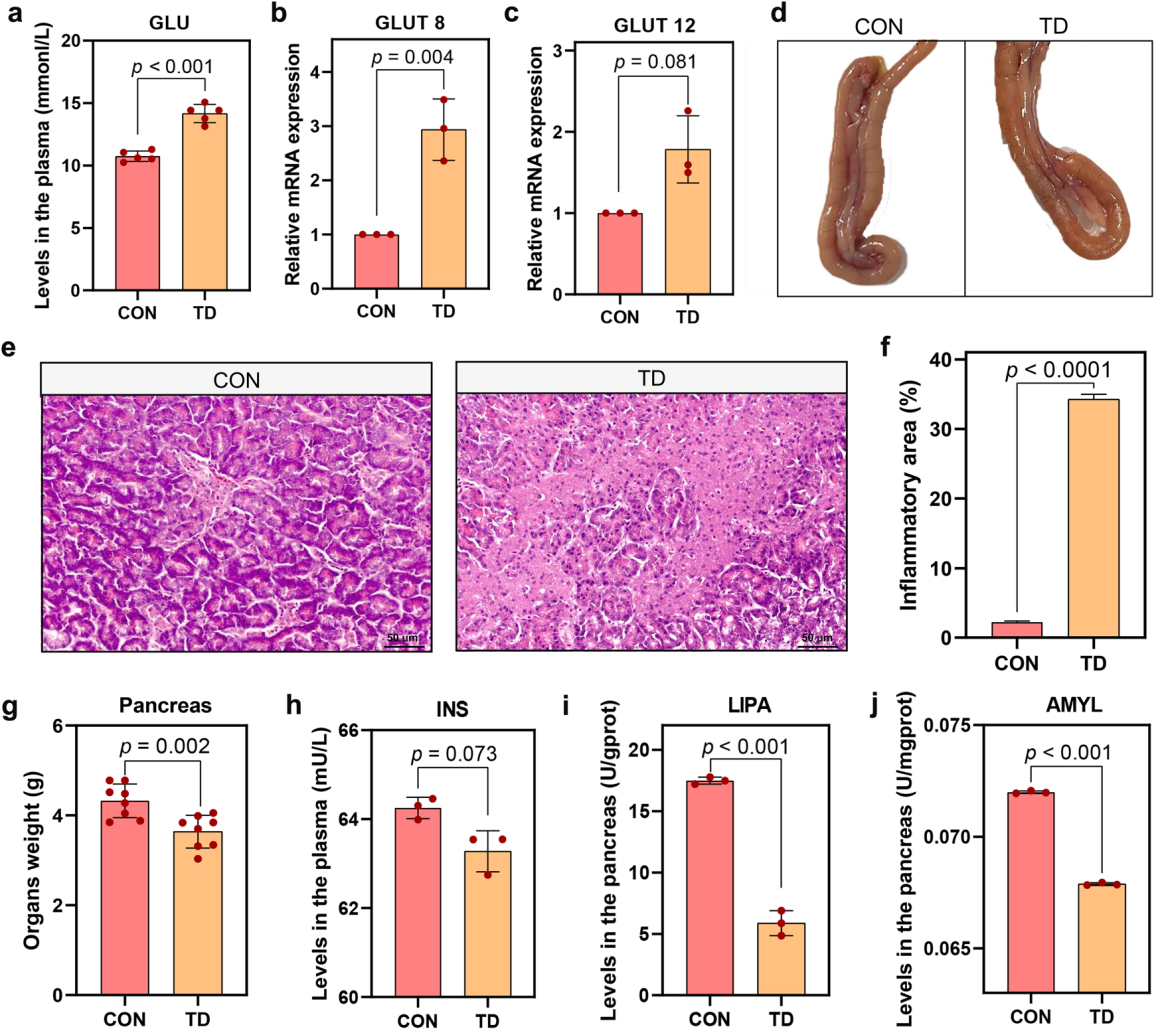
Effects of TD on blood GLU levels and the pancreas in broilers. **a** Plasma GLU levels (*n* = 5 broilers per group). **b**, **c** *GLUT8* and *GLUT12* mRNA expression levels in the pancreas (*n* = 3 broilers per group). **d** Pancreas morphology. **e** Representative images of HE-stained sections of the pancreas (scale bars, 50 μm). **f** Quantitative analysis of the percentage of inflammatory infiltration area in (**e**). **g** The weight of the pancreas. **h** INS levels in the pancreas. **i**, **j** LIPA and AMYL levels in the pancreas (*n* = 3 broilers per group). GLU glucose, INS insulin, AMYL, α-amylase, LIPA lysosomal acid lipase A. A significant difference was indicated as a *p* value less than 0.05 calculated using a two-tailed unpaired Student’s *t* test. The data are presented as means ± SD.

We monitored the blood glucose (GLU) levels in normal and TD broilers on day 7 to determine the relationship between GLU levels and TD in broilers. As shown in Fig. 1a, the GLU levels in TD broilers were significantly higher than those in the CON group (*p* < 0.001). In addition, the mRNA expression levels of glucose transporter 8 (GLUT8, *p* = 0.004) and glucose transporter 12 (GLUT12, *p* = 0.081) were elevated in the pancreases of broilers from the TD group compared with the CON group (Fig. 1b, c).

The pancreas is the main organ that secretes hormones to regulate blood GLU levels. Morphological changes in this organ were the first concern (Fig. 1d). Pancreatic tissue from broilers in the TD group showed a dull surface, reduced volume, narrower width, thinner girth and hard texture, while the pancreatic tissue from the CON group showed a normal grey-red colour with a uniform texture and full shape. In addition, the pancreatic weight in the TD group was significantly lighter than that in the CON group (Fig. 1g). A subsequent pathological examination of the tissue showed that the pancreatic acini in the TD group were atrophic and necrotic, with large fibrous structures in the dissipated part of the necrotic tissue (Fig. 1e, f). Next, the secretory function of the pancreas was assessed, and the levels of insulin (INS) in plasma and lysosomal acid lipase A (LIPA) and α-amylase (AMYL) in the pancreas were significantly reduced (*p* = 0.073, *p* < 0.001, and *p* < 0.001, respectively) in the TD group compared with the CON group (Fig. 1h–j). Based on these results, blood GLU levels in TD broilers are significantly elevated, and pancreatic damage may be one of the main factors responsible for the elevation in GLU levels.

### The GM modulated the susceptibility of TD broilers to changes in blood GLU levels

**Fig. 2:**
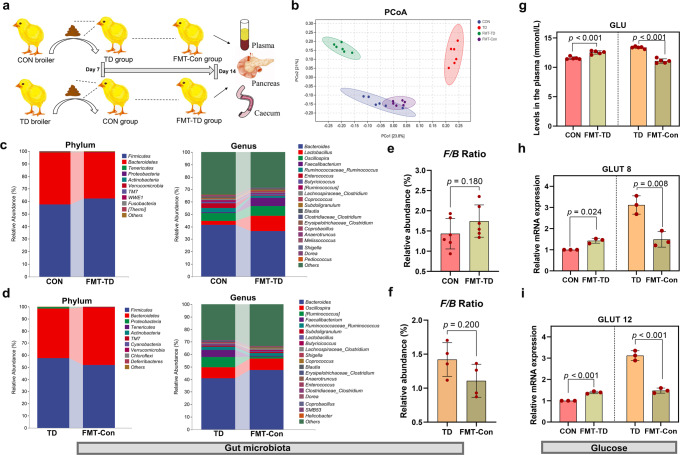
Establishment of the FMT model and the effect of GM on blood GLU levels in broilers. **a** Establishment of the FMT model. **b** Bray–Curtis distance-based PCoA estimates for the GM of the CON (blue), TD (red), FMT-TD (green) and FMT-Con (purple) groups. **c**, **d** The top ten phyla in the GM in terms of abundance. The top 20 most abundant GM at the genus level. **e**, **f** Differential abundances of F/B ratios by phylum. **g** GLU levels in plasma (*n* = 5 broilers per group). **h**, **i** *GLUT8* and *GLUT12* mRNA expression levels in the pancreas (*n* = 3 broilers per group). FMT faecal microbiota transplantation, GLU glucose. A significant difference was defined as a *p* value less than 0.05 calculated using a two-tailed unpaired Student’s *t* test. The data are presented as the means ± SD.

The pancreas is bound to the intestine via the pancreatic duct. By performing the faecal microbiota transplantation (FMT) experiment, we assessed the effect of FMT on the GM and blood GLU levels in broilers to determine whether GM is involved in regulating blood GLU levels (Fig. 2a). The α-diversity analysis using different indices, including the Chao1 and observed species indices, showed increasing trends in the indices of GM in the FMT-Con group compared to the TD group (*p* = 0.15 and *p* = 0.083, respectively). In contrast, the FMT-TD group showed decreasing trends compared to the CON group (*p* = 0.87 and *p* = 0.42, respectively) (Supplementary Fig. 1a). We observed a clear separation between the TD and CON groups using Bray–Curtis distance-based nonmetric multidimensional scaling (NMDS), principal component analysis (PCA) and principal coordinate analysis (PCoA) (Supplementary Fig. 1b, c; Fig. 2b). However, the FMT-TD group was closer to the TD group in terms of distance on the vertical coordinate compared with the CON group, and the FMT-Con group almost completely overlapped with the CON group in the PCoA analysis of the FMT experiment (Supplementary Fig. 1b, c; Fig. 2b). In addition, evaluations at the phylum and genus levels showed that FMT changed the microbiota compositions of the CON and TD groups (Fig. 2c, d). An increasing trend in the abundance of Firmicutes and the Firmicutes/Bacteroidetes ratio (F/B ratio) was observed in the FMT-TD group compared to the CON group (*p* = 0.180) (Supplementary Fig. 1d; Fig. 2e). Conversely, a decreasing trend in the abundance of Firmicutes and the F/B ratio was observed in the FMT-Con group compared to the TD group (*p* = 0.200) (Fig. 2f). These data suggest that FMT effectively reverses the GM dysfunction in TD broilers.

The changes in blood GLU-related indicators are shown in Fig. 2g–i. Compared with the CON group, the FMT-TD group of broiler chickens showed higher levels of GLU (*p* < 0.001) in the plasma (Fig. 2g) and obviously increased mRNA expression levels of *GLUT8* and *GLUT12* (*p* = 0.024 and *p* < 0.001, respectively) in the pancreas (Fig. 2h, i). Moreover, the plasma GLU levels and pancreatic *GLUT8* and *GLUT12* mRNA levels in the FMT-Con group broilers were significantly reduced compared with those in the TD group (*p* < 0.001, *p* = 0.008, and *p* < 0.001, respectively; Fig. 2g–i). Thus, GM is associated with changes in blood GLU levels in TD broilers.

### Impaired gut barrier function in TD broilers

**Fig. 3:**
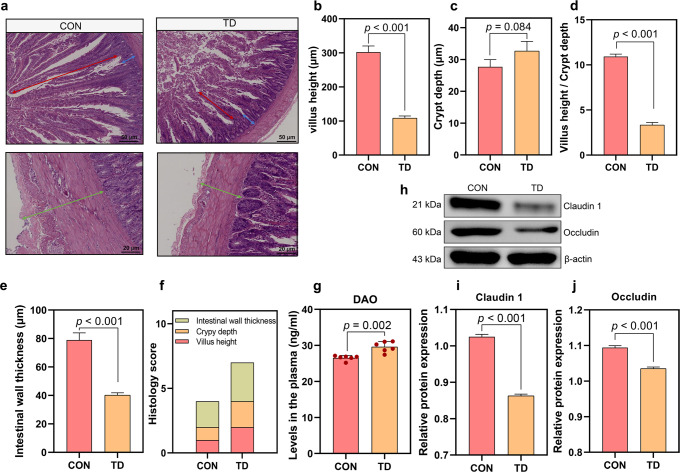
Duodenal morphology and mucosal barrier function of broiler chickens in the TD and CON groups. **a** Representative images of HE-stained sections of the duodenum. Red arrow, villus height. Blue arrow, crypt depth. Cyan arrow, intestinal wall thickness (scale bars: top, 50 µm; bottom, 20 µm). **b**–**d** Statistical analyses of the villus height, crypt depth and the ratio of villus height to crypt depth. **e** Statistical analysis of the intestinal wall thickness. **f** Stacked bar graph showing the differences in the total histology scores and individual histological criteria scores between the two groups. **g** Plasma DAO levels (*n* = 6 broilers per group). **h**–**j** Western blots showing Claudin 1 and Occludin expression in the duodenum. DAO diamine oxidase. A significant difference was defined as a *p* value less than 0.05 calculated using a two-tailed unpaired Student’s *t* test. The data are presented as the means ± SD.

The gut barrier was first assessed by performing a pathological examination to investigate the specific alterations in the gut involved in the regulation of blood GLU levels (Fig. 3a). The height of the intestinal villus (*p* < 0.001), the ratio of the intestinal villus to crypt depth (V/C, *p* < 0.001) and the thickness of the intestinal walls (*p* < 0.001) were decreased significantly (Fig. 3b, d, e), while the crypt depth was deeper (*p* = 0.084) in the TD group than in the CON group (Fig. 3c). The overall histology score also showed that the TD group had a severely compromised gut barrier (Fig. 3f). The plasma levels of diamine oxidase (DAO, *p* = 0.002) were significantly increased in the TD group compared with the CON group (Fig. 3g). The western blot results further showed markedly lower levels of the Claudin 1 (*p* < 0.001) and Occludin proteins (*p* < 0.001) in the TD group than those in the CON group (Fig. 3h–j). These findings reveal that the intestine is compromised, as evidenced by alterations in its morphological structure, permeability and mucosal barrier function in TD broilers.

### Disturbance of the GM structure and imbalance of the microbiota ratio in TD broilers

By analysing the 16 S rRNA gene sequences of broiler caecal contents in the TD and CON groups, we determined whether the overall structure of GM changed in response to blood GLU levels. The α-diversity analysis using different indices, including the Chao1, Shannon, Simpson and observed species indices (all *p* = 0.0039), showed significant differences in the indices of GM in the TD group compared to the CON group (Supplementary Fig. 2a). Bray–Curtis distance-based NMDS, PCA, and PCoA displayed differences in the clustering of the GM community between the CON and TD groups, indicating that the gut microbial structure was significantly different (Supplementary Fig. 2b–d).

**Fig. 4:**
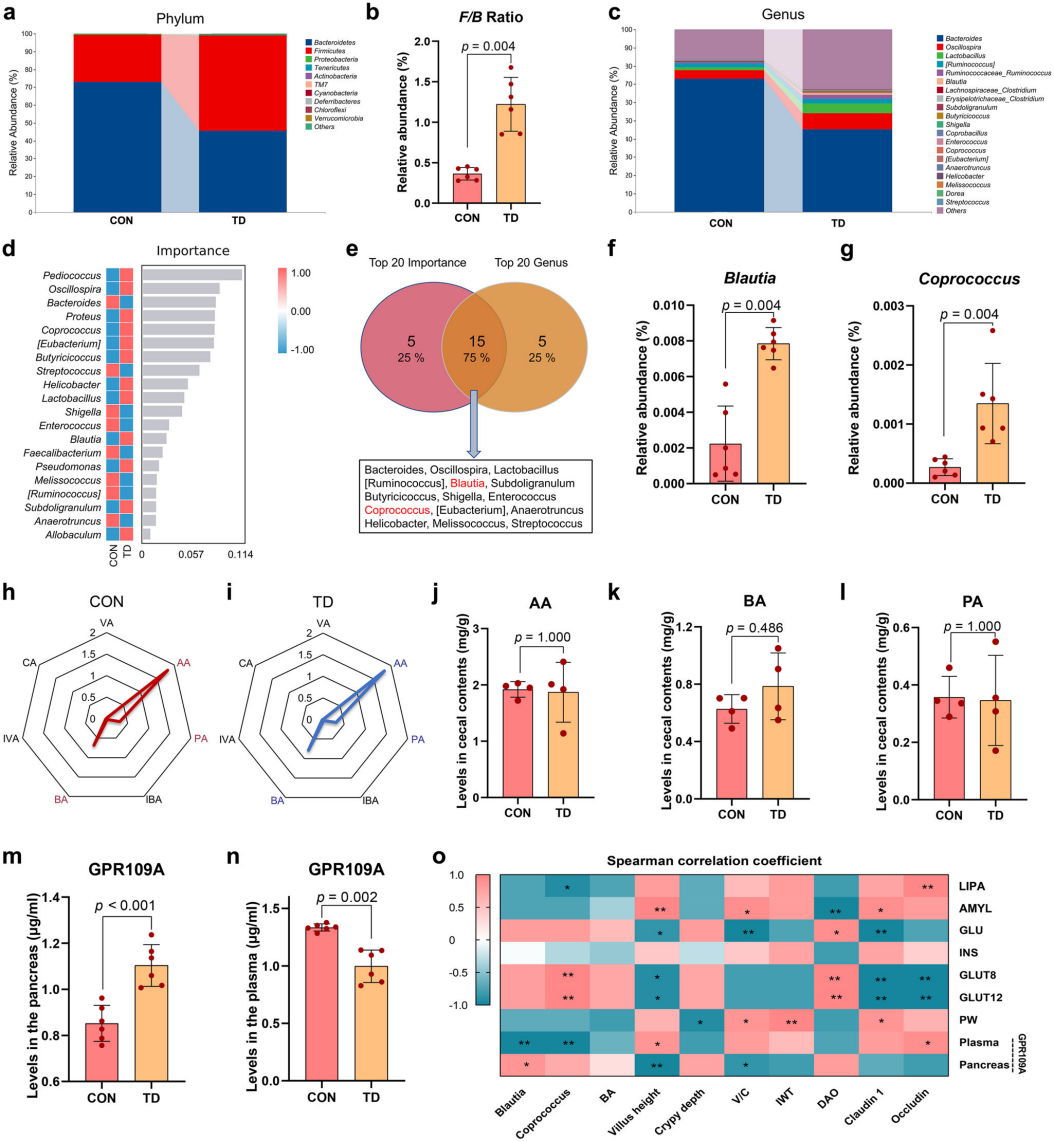
Differences in GM abundance and SCFA levels in the TD and CON groups. **a** The top ten phyla of the GM in terms of abundance. **b** Differential abundances of F/B ratios by phylum. **c** The top 20 most abundant GM components at the genus level. **d** Genus-level analysis of the GM using the random forest model. The abscissa indicates the importance of species to the classifier model, while the ordinate indicates the taxa name at the genus level. From top to bottom, the importance of species to the model decreases. **e** Venn diagrams of two sets of the top 20 species in terms of abundance and importance. **f**, **g** Differences in the abundances of GM components (*Blautia* and *Coprococcus*, respectively) at the genus level. **h**, **i** Radar chart showing the enrichment of seven SCFAs in the CON (red) and TD (blue) groups. Each corner represents an SCFA, and the length of the line from the centre to the corner represents the abundance of SCFAs. **j**–**l** The AA, BA, and PA levels in caecal contents. **m**, **n** GPR109A levels in the pancreas and plasma (*n* = 6 broilers per group). **o** Spearman’s analysis of the correlations between gut-related metrics and glucose-related parameters. Red, positive correlation. Blue, negative correlation. F/B firmicutes/bacteroidetes, AA acetic acid, BA butyric acid, CA caproic acid, IBA isobutyric acid, INS insulin, IVA isovaleric acid, VA valeric acid, GPR109A G-protein-coupled receptor 109A. A significant difference was defined as a *p* value less than 0.05 calculated using the Mann‒Whitney *U* test. The data are presented as the means ± SD.

Bacterial communities in broiler caecal contents were dominated by Firmicutes, Bacteroidetes and Proteobacteria at the phylum level (Fig. 4a). The proportion of the phylum Bacteroidetes (45.46%) in the TD group was decreased compared with that in the CON group (72.93%; *p* = 0.004), but the proportion of the phylum Firmicutes (53.62%) in the TD group was increased compared with that in the CON group (26.30%; Supplementary Fig. 2e; *p* = 0.004). The F/B ratio also increased significantly (Fig. 4b, *p* = 0.004). In addition, the genera that were differentially enriched between the CON and TD groups were *Bacteroides, Oscillospira*, *Lactobacillus*, *[Ruminococcus]*, *Ruminococcaceae Ruminococcus* and *Blautia* (Fig. 4c).

Then, a random forest analysis was conducted to screen the top 20 important species, and the species with the highest importance included *Pediococcus*, *Oscillospira*, *Bacteroides*, *Proteus*, and *Coprococcus* (Fig. 4d). Furthermore, a Venn diagram of the top 20 species in terms of abundance and importance was constructed, and 15 species were identified, such as *Bacteroides*, *Blautia*, and *Coprococcus* (Fig. 4e). In addition, the abundances of *Blautia* and *Coprococcus* in the TD group were significantly higher than those in the CON group (Fig. 4f, g; *p* = 0.004 and *p* = 0.004, respectively). Our findings revealed that changes in the structure of the GM might contribute to the production of high blood GLU levels.

### Differences in SCFA metabolism in the gut and differential expression of GPR109A in the pancreas of TD broilers

Short-chain fatty acid (SCFA) sequencing analysis was subsequently performed to determine the effect of GM metabolites on the regulation of blood GLU levels. The radar chart of the results showed that propionic acid (PA), acetic acid (AA) and butyric acid (BA) were most abundant in the TD and CON groups (Fig. 4h, i). Compared with the CON group, the increasing trend of BA (*p* = 0.486) levels in the TD group was more evident than changes in the levels of AA (*p* = 0.100) and PA (*p* = 0.100) (Fig. 4j–l). Based on this finding, BA may be the main GM metabolite that affects blood GLU levels. We aimed to further reveal the relationship between BA and blood GLU levels by measuring the levels of the BA signature receptor G-protein-coupled receptor 109A (GPR109A) in the pancreas and plasma (Fig. 4m, n). The levels of GPR109A (*p* < 0.001) in the pancreas were elevated in the TD group compared with the CON group (Fig. 4m). Conversely, the plasma levels of GPR109A (*p* = 0.002) were considerably lower in the TD group than in the CON group (Fig. 4n). Therefore, BA exerts a blood GLU-lowering effect by binding to the receptor GPR109A in the pancreas of TD chickens.

Spearman’s correlation analysis was conducted to investigate the correlations between blood GLU levels and gut-related indices (Fig. 4o). The findings revealed that the pancreatic GPR109A content was positively correlated with *Blautia* abundance (*r* = 0.829 and *p* = 0.042) and negatively correlated with the villus height (*r* = −0.943 and *p* = 0.005). The plasma GPR109A content was negatively correlated with *Blautia* abundance (*r* = −0.943 and *p* = 0.005) and positively correlated with the villus height (*r* = 0.829 and *p* = 0.042). *GLUT12* and *GLUT8* mRNA expression levels were positively correlated with *Coprococcus* abundance (*r* = 0.941 and *p* = 0.005) and DAO levels (*r* = 0.941 and *p* = 0.005) and negatively correlated with the villus height (*r* = −0.880 and *p* = 0.021), Claudin 1 protein expression (*r* = −0.941 and *p* = 0.005) and Occludin protein expression (*r* = −0.941 and *p* = 0.005). GLU levels were negatively correlated with the villus height (*r* = −0.829 and *p* = 0.042), V/C (*r* = −0.943 and *p* = 0.005) and Claudin 1 protein expression (*r* = −0.943 and *p* = 0.005), while AMYL levels were positively correlated with these factors (*r* = 0.943, 0.886 and 0.886, *p* = 0.005, 0.019 and 0.019, respectively). These results suggest that blood GLU levels in TD broilers are strongly correlated with gut-related markers.

### TFRD improves the gut barrier in TD broilers

**Fig. 5:**
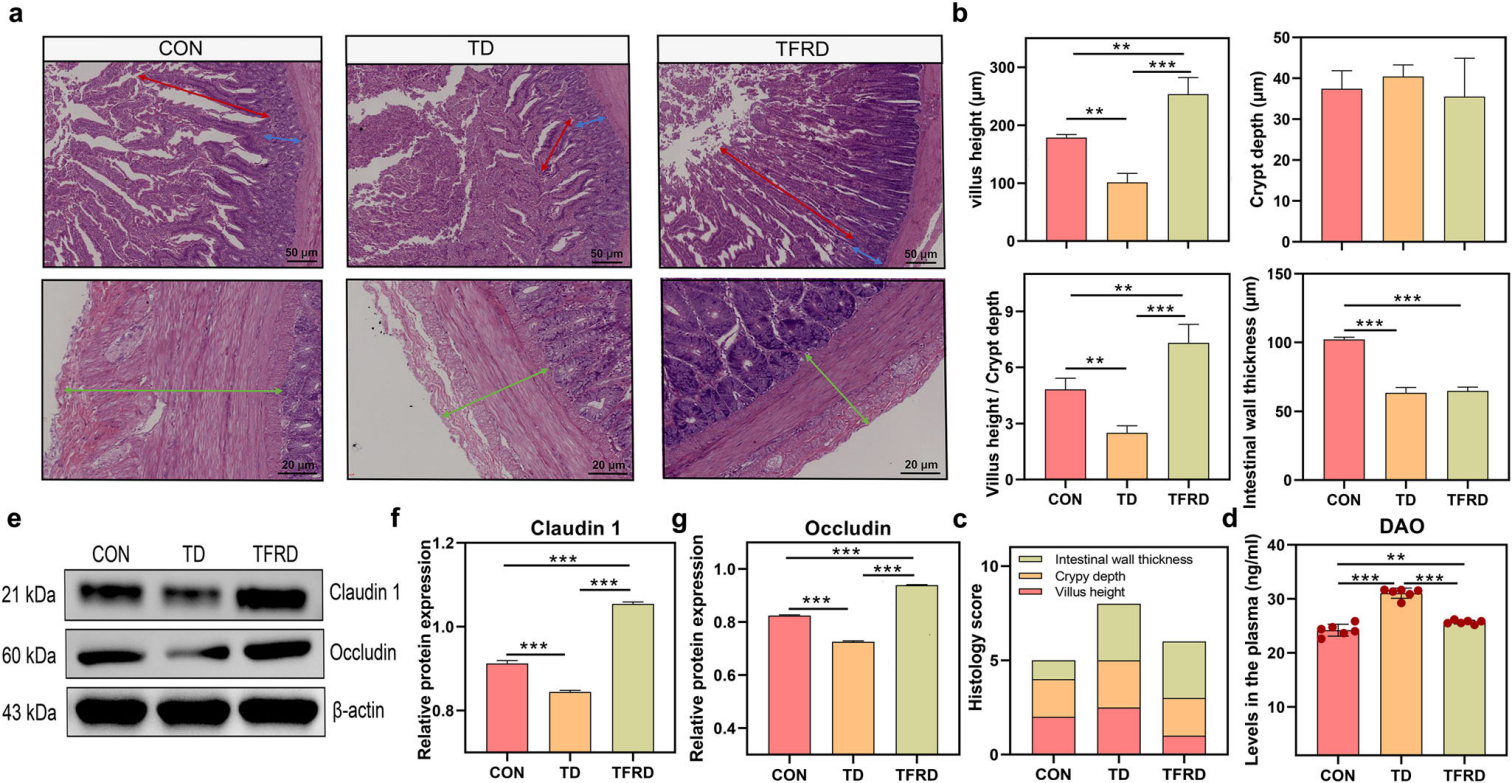
Duodenal morphology and mucosal barrier function after TFRD supplementation. **a** Representative images of HE-stained sections of the duodenum. Red arrow, villus height. Blue arrow, crypt depth. Cyan arrow, intestinal wall thickness (scale bars: top, 50 µm; bottom, 20 µm). **b** Statistical analyses of the villus height, crypt depth, the ratio of villus height to crypt depth and intestinal wall thickness. **c** Stacked bar graph showing the differences in the total histology scores and individual histological criteria scores between the three groups. **d** Plasma DAO levels (*n* = 6 broilers per group). **e**–**g** Western blots showing Claudin 1 and Occludin expression in the duodenum. *p < 0.05, **p < 0.01, and ***p < 0.001. One-way ANOVA with LSD post hoc test. The data are presented as the means ± SD.

TD broilers were fed total flavonoids from *Rhizoma Drynariae* (TFRD) for two weeks to verify the involvement of the gut barrier in modulating blood GLU levels. The results of the gut pathological examination are shown in Fig. 5a. TFRD treatment significantly increased the villus height (*p* < 0.001) and the V/C ratio (*p* < 0.001) in TD broilers, but significant differences in crypt depth and intestinal wall thickness were not observed (Fig. 5b). The gut histology score also showed that TFRD supplementation reduced gut barrier damage in TD broilers (Fig. 5c). In addition, plasma DAO levels were highly significantly reduced (*p* < 0.001) in the TFRD group compared with the TD group (Fig. 5d). Subsequently, intestinal tight junction proteins were analysed using western blotting (Fig. 5e). The protein expression levels of Claudin 1 (*p* < 0.001) and Occludin (*p* < 0.001) were highly significantly upregulated in the TFRD group compared with the TD group (Fig. 5f, g). These findings showed that the restoration of gut barrier function and permeability reduces the production of high blood GLU levels.

### TFRD improves the GM in TD broilers

**Fig. 6:**
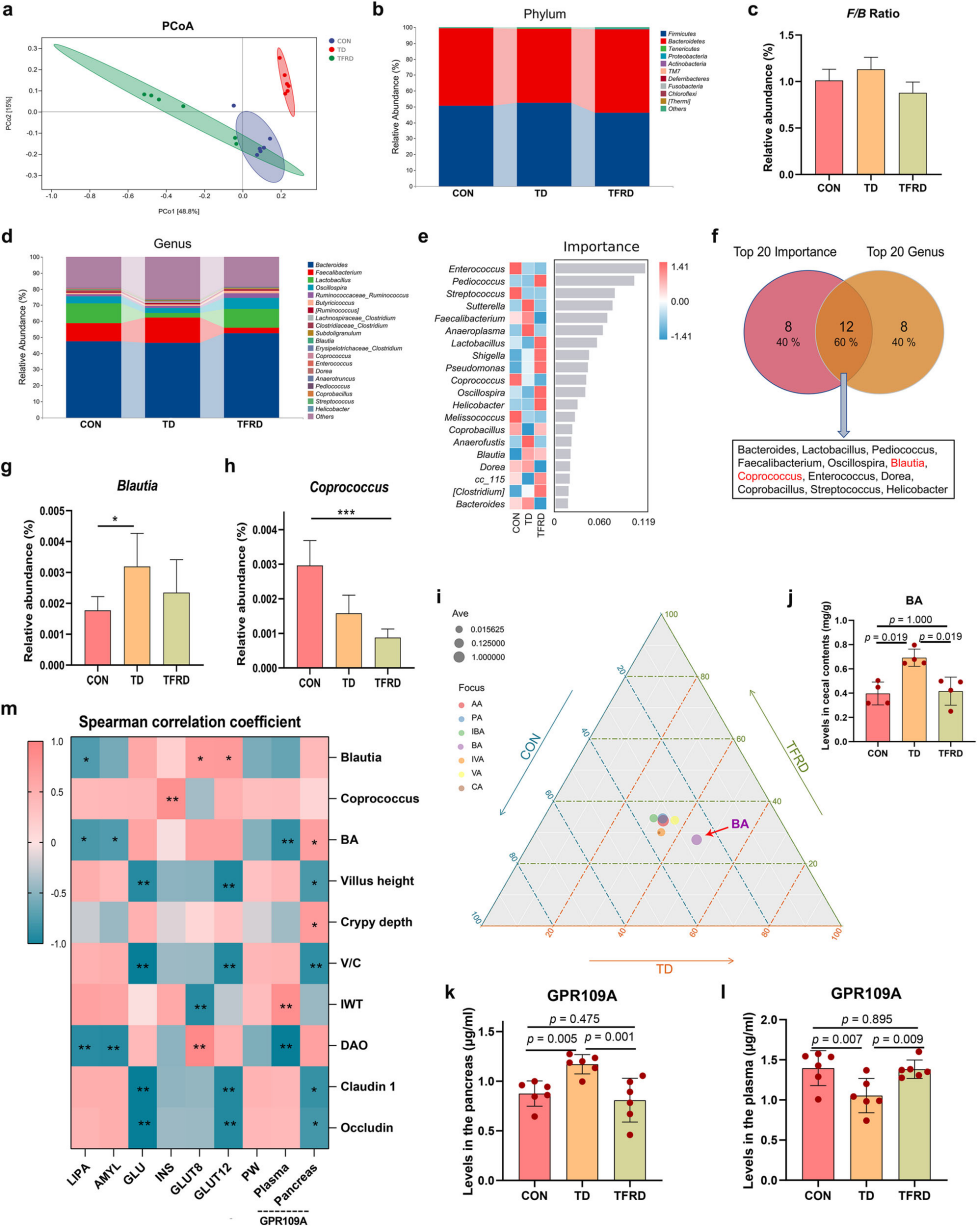
Effects of the GM and SCFAs on GLU levels mediated by GPR109A. **a** Bray–Curtis distance-based PCoA analysis of the GM in the CON (blue), TD (red) and TFRD (green) groups of broiler chickens. **b** The top ten phyla of the GM in terms of abundance. **c** Differential abundances of firmicutes/bacteroidetes (F/B) ratios by phylum. **d** The top 20 most abundant GM constituents at the genus level. **e** Genus-level analysis of the GM using the random forest model. The abscissa indicates the importance of species to the classifier model, while the ordinates indicate the taxon names at the genus level. From top to bottom, the importance of species to the model decreases. **f** Venn diagrams of two sets of the top 20 species in terms of abundance and importance. **g**, **h** Differences in the abundances of GM components (*Blautia* and *Coprococcus*, respectively) at the genus level. **i** Ternary diagram showing the enrichment of seven SCFAs in the CON, TD and TFRD groups. Each side of the triangle represents a group. The closer the vertical distance from the bubble to the edge, the larger the proportion in the group. The size of the bubble indicates the SCFA abundance. **j** BA levels in caecal contents. **k**, **l** GPR109A levels in the pancreas and plasma (*n* = 6 broilers per group). **m** Spearman’s analysis of the correlations between gut-related metrics and GLU-related parameters. Red, positive correlation. Blue, negative correlation. *p < 0.05, **p < 0.01, and ***p < 0.001. Kruskal‒Wallis one-way ANOVA. The data are presented as the means ± SD.

The GM of broilers in the TFRD group also changed after 2 weeks of supplementation. The results of Bray–Curtis distance-based PCoA showed that the composition of the GM of broilers in the TD group after supplementation with TFRD was similar to that in the CON group (Fig. 6a). The Firmicutes, Bacteroidetes and Tenericutes phyla were the most abundant phyla detected in the caecal contents (Fig. 6b). The proportion of the phylum Bacteroidetes (52.65%) in the TFRD group was higher than that in the TD group (46.57%; Supplementary Fig. 2f), but the abundances of the phyla Firmicutes (46.05%) and Tenericutes (0.087%) in the TFRD group were decreased compared with those in the TD group (52.50% and 0.323%, respectively). Furthermore, the F/B ratio in the TFRD group was decreased by 22.44% compared with that in the TD group (*p* > 0.05, Fig. 6c). Additionally, a comparison of the genera with differences in abundance between the CON, TD and TFRD groups revealed that *Bacteroides, Faecalibacterium*, *Lactobacillus*, *Oscillospira*, *Ruminococcaceae Ruminococcus*, *Butyricicoccus* and *[Ruminococcus]* were distinctly enriched (Fig. 6d).

Based on the random forest analysis, the top 20 crucial species included *Enterococcus*, *Pediococcus*, *Streptococcus*, *Sutterella* and *Faecalibacterium* (Fig. 6e). The Venn diagram of the top 20 species ranked by abundance and importance identified 12 species, including *Bacteroides*, *Lactobacillus*, *Blautia* and *Coprococcus*. (Fig. 6f). Among these, the abundances of *Blautia* (*p* > 0.05) and *Coprococcus* (*p* > 0.05) in the TFRD group were markedly decreased by 26.60% and 44.34%, respectively, compared with their abundances in the TD group (Fig. 6g, h). Based on these results, restoring gut dysbiosis reduces high blood GLU production.

### TFRD improves the levels of the gut metabolites SCFAs in TD broilers

The ternary diagram showed that of the seven SCFAs, BA was the closest to the TD and TFRD groups (Fig. 6i). A subsequent quantitative analysis showed that BA levels were significantly decreased in the TFRD group (*p* = 0.019) compared with the TD group (Fig. 6j). An analysis of the BA receptor GPR109A indicated that the levels of GPR109A (*p* = 0.001) in the pancreas were lower in the TFRD group than in the TD group (Fig. 6k). Conversely, the plasma levels of GPR109A (*p* = 0.009) were elevated in the TFRD group compared with the TD group (Fig. 6l). Thus, BA reduces blood GLU levels by binding to the receptor GPR109A in the pancreas of TD broilers.

Spearman’s correlation analysis was conducted to investigate the correlations between blood GLU levels and gut-related indices (Fig. 6m). The findings indicated that the pancreatic GPR109A activities were positively correlated with BA levels (*r* = 0.683, *p* = 0.042), while the plasma GPR109A activities were negatively correlated with BA levels (*r* = −0.800, *p* = 0.010). *GLUT12* and *GLUT8* mRNA expression levels were positively correlated with *Blautia* abundance (*r* = 0.730 and 0.712, *p* = 0.026 and 0.031, respectively). INS levels were positively correlated with *Coprococcus* abundance (*r* = 0.833, *p* = 0.005). GLU levels were negatively correlated with the villus height (*r* = −0.085, *p* = 0.004), the V/C ratio (*r* = −0.967, *p* < 0.001) and Claudin 1 protein expression (*r* = −0.967, *p* < 0.001). LIPA and AMYL levels were negatively correlated with BA levels (*r* = −0.717 and −0.683, *p* = 0.030 and 0.042, respectively) and DAO levels (*r* = −0.833 and −0.850, *p* = 0.005 and 0.004, respectively). These results indicated that blood GLU levels in TD broilers are strongly correlated with gut-related markers.

### Inhibition of PI3K/AKT/VEGFA expression in TGPs of TD broilers

**Fig. 7:**
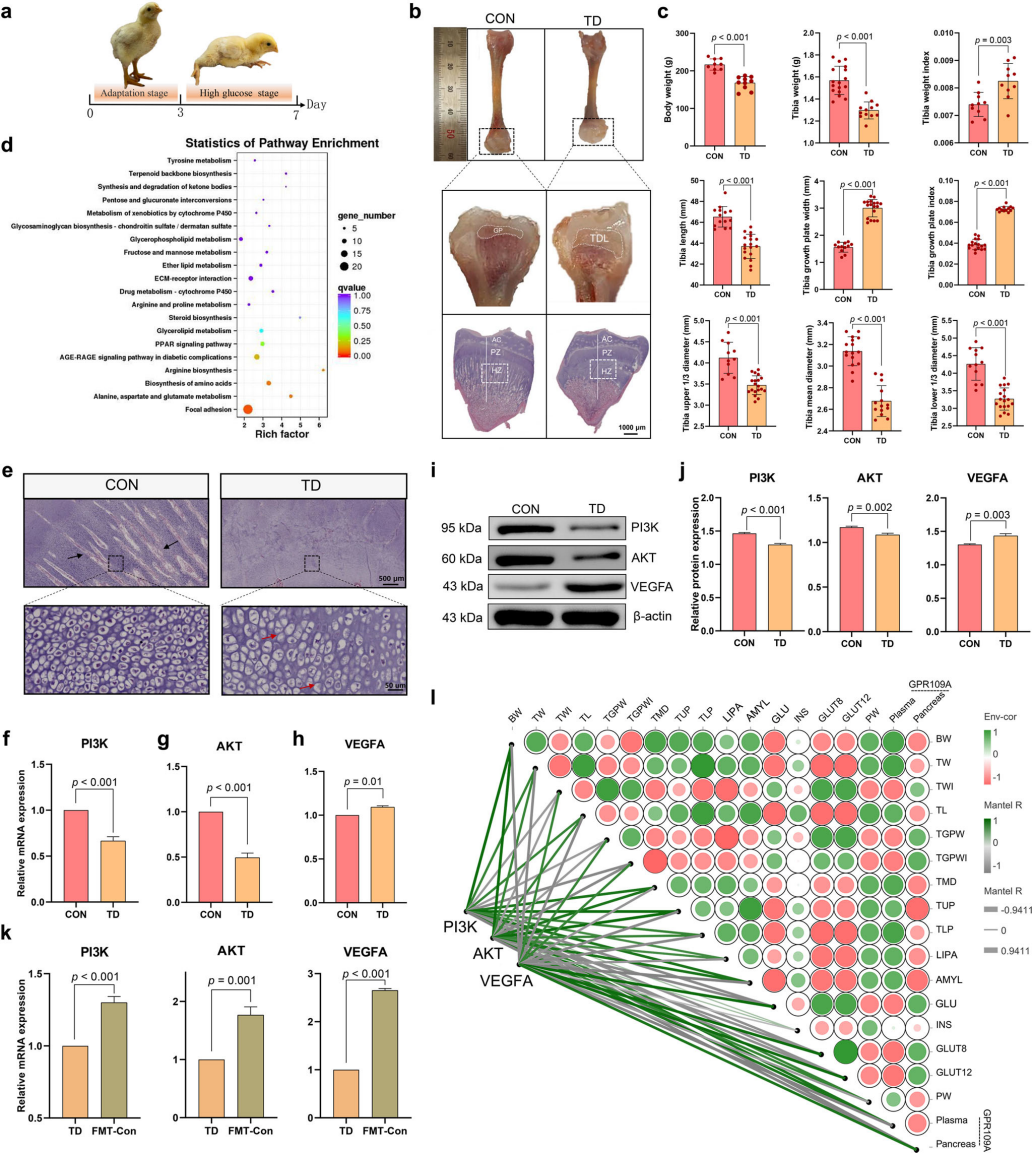
Effects of PI3K, AKT and VEGFA expression on tibial growth in the TD and CON groups. **a** Clinical characteristics of broiler chickens. **b** Morphological observation of the tibia and the HE staining of the TGPs (scale bars, 1000 µm). **c** Histogram showing body weight and tibia-related indicators. **d** Analysis of potential pathways based on DEGs in the tibia from the CON and TD groups of chickens. The bubble size indicates the number of DEGs enriched in this pathway, and points with different gradations of colour (from red to blue) represent the scope of the *p* value. **e** Representative images of HE-stained sections of TGPs. Black arrow, blood vessel. Red arrow, nuclear dissolution and nuclear pyknosis (scale bars: top, 500 µm; bottom, 50 µm). **f**–**h** The mRNA expression levels of *PI3K*, *AKT* and *VEGFA* in the TGPs (*n* = 3 broilers per group). **I**, **j** PI3K, AKT and VEGFA protein levels in the TGPs (n = 3 broilers per group). (**k**) The mRNA expression levels of *PI3K*, *AKT* and *VEGFA* in the TD and FMT-Con groups (*n* = 3 broilers per group). **l** The heatmap on the right shows the results of Spearman’s analysis of the correlations between GLU-related indicators and bone parameters. Green, positive correlation. Red, negative correlation. The heatmap on the left shows the results of Spearman’s analysis of the correlations of PI3K, AKT and VEGFA levels with glucose-bone indicators. Green, positive correlation. Grey, negative correlation. GP growth plate, TDL tibial dyschondroplasia lesion, AC articular cartilage, HZ hypertrophic zone, PZ proliferation zone, PI3K phosphatidylinositol-3-kinase, AKT protein kinase B, VEGFA vascular endothelial growth factor. A significant difference was defined as a *p* value less than 0.05 calculated using a two-tailed unpaired Student’s *t* test. The data are presented as means ± SD.

Experiments showed that TD broilers were in the high glucose stage from day 3 to day 7 and had obvious clinical symptoms of TD, with split legs and an inability to stand (Fig. 7a). The morphological observations revealed that the tibias of broilers in the TD group were significantly hypoplastic and exhibited a short and thin tibial backbone. Moreover, transparent white cartilage thrombi without calcification and vascularisation appeared in the TGP of the TD group, and the white cartilage thrombus area was significantly thickened, occupying most of the section of the dry pulp end (Fig. 7b). The results of the quantitative bone histomorphologic analysis are shown in Fig. 7c. Further pathological observations of the tibial epiphysis showed an obvious reduction in the vascularity of the hypertrophic zone and delayed calcification in the TD group (Fig. 7b).

We used the differentially expressed tibial genes identified using a transcriptome sequencing analysis and mapped them to the KEGG database for functional clustering analysis to explore the potential regulatory mechanisms of tibial damage in TD broiler chickens; we observed significant enrichment in the focal adhesion pathway (Fig. 7d). The blood vessels in the TGP area were more abundant, the osteoblast distribution was relatively dense, and the osteoblast structure was relatively regular in the CON group (Fig. 7e). In the TD group, no invasion of blood vessels was detected in the corresponding area of the tibia, the distribution of osteoblasts in the hypertrophic area was more dispersed, and most osteoblasts showed nuclear consolidation and nucleolysis. In addition, the results of quantitative real-time PCR revealed that the mRNA expression levels of phosphatidylinositol-3-kinase (PI3K, *p* < 0.001) and protein kinase (AKT, *p* < 0.001) in the TGP were extremely significantly lower in the TD group than in the CON group (Fig. 7f, g), but *VEGFA* mRNA expression (*p* = 0.001) was higher in the TD group (Fig. 7h). Western blot results showed markedly lower levels of the PI3K (*p* < 0.001) and AKT proteins (*p* = 0.002) in the TD group than those in the CON group, and the VEGFA protein (*p* = 0.003) was expressed at higher levels in the TD group (Fig. 7i, j). After FMT, the mRNA expression levels of *PI3K* (*p* < 0.001), *AKT* (*p* = 0.001) and *VEGFA* (*p* < 0.001) in the TD group were significantly lower than those in the FMT-Con group (Fig. 7k). These results indicated that high blood GLU levels promote skeletal damage in TD broilers via the PI3K/AKT/VEGFA signalling pathway.

Furthermore, Spearman’s correlation analysis was performed to investigate the correlation between GLU-related indicators and bone-related parameters and the correlation between the PI3K/AKT/VEGFA signalling pathway, blood GLU-related indicators and bone-related parameters (Fig. 7l). The dynamic network heatmap showed that GLU and pancreatic GPR109A levels were negatively correlated with the tibia upper 1/3 diameter (TUP) (*r* = −0.886 and −0.943, *p* = 0.019 and 0.004, respectively), while AMYL levels (*r* = 0.943, *p* = 0.004) were positively correlated with TUP. The *GLUT8* (*r* = 0.880, *p* = 0.021) and *GLUT12* mRNA expression levels (*r* = 0.880, *p* = 0.021) were positively correlated with the tibia growth plate width (TGPW). Furthermore, the *PI3K* and *AKT* mRNA expression levels were significantly negatively correlated with the bone GLU levels (*r* = −0.880, *p* = 0.021, respectively) and the TGPW index (TGPWI) (*r* = −0.880, *p* = 0.021, respectively). Conversely, the *PI3K* and *AKT* mRNA expression levels were significantly positively correlated with AMYL levels (*r* = 0.880, *p* = 0.021, respectively), TUP (*r* = 0.941, *p* = 0.005, respectively) and the tibia middle diameter (TMD) (*r* = 0.880, *p* = 0.021, respectively). The correlations between *VEGFA* mRNA expression levels and glucose-bone-related indices were opposite to those of the *PI3K* and *AKT* mRNA expression levels and glucose-bone-related indices. Based on these results, PI3K/AKT/VEGFA signalling is involved in the mechanism regulating blood GLU levels and gut-related markers in TD broilers.

### TFRD reduces blood GLU levels and suppresses bone damage in TD broilers by regulating the PI3K/AKT/VEGFA pathway

**Fig. 8:**
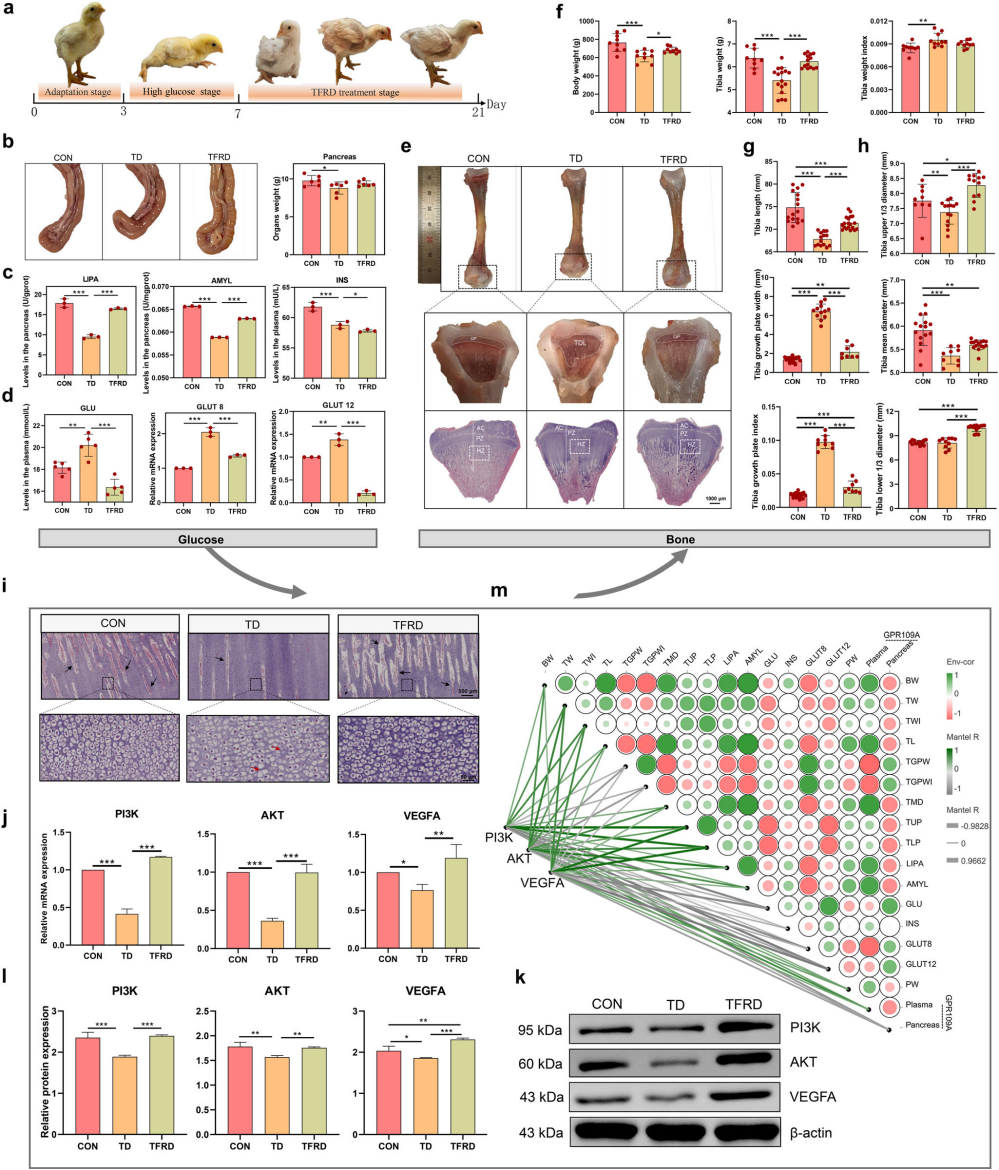
The effects of GLU on tibial growth are mediated by regulating the PI3K/AKT/VEGFA signalling pathway. **a** Clinical characteristics of broiler chickens. **b** Pancreas morphology and weight. **c** The LIPA, AMYL and INS levels in the pancreas (*n* = 3 broilers per group). **d** Plasma GLU levels. *GLUT8* and *GLUT12* mRNA expression levels in the pancreas (*n* = 3 broilers per group). **e** Morphological observation of the tibia and the HE staining of the TGPs (scale bars, 1000 µm). **f**–**h** Histogram showing the body weight and tibia-related indicators. **i** Representative images of HE-stained sections of TGPs. Black arrow, blood vessel. Red arrow, nuclear dissolution and nuclear pyknosis. (scale bars: top, 500 µm; bottom, 50 µm). **j** *PI3K*, *AKT* and *VEGFA* mRNA expression levels in the TGPs (*n* = 3 broilers per group). **k**, **l** PI3K, AKT and VEGFA protein levels in the TGPs (*n* = 3 broilers per group). **m** The heatmap on the right shows the results of Spearman’s analysis of the correlations between GLU-related indicators and bone parameters. Green, positive correlations. Red, negative correlations. The heatmap on the left shows the results of Spearman’s analysis of the correlations of PI3K, AKT and VEGFA levels with glucose-bone indicators. Green, positive correlation. Grey, negative correlation. *p < 0.05, **p < 0.01, and ***p < 0.001. One-way ANOVA with LSD post hoc test. The data are presented as the means ± SD.

Chickens supplemented with TFRD showed significant improvements in clinical symptoms after 2 weeks, and were able to stand on both feet and walk slowly (Fig. 8a). Evaluations of pancreatic morphology revealed that the pancreatic tissue of the TFRD group was a normal grey-red colour with a uniform texture and full shape compared with the TD group (Fig. 8b). Similarly, the pancreas weight in the TFRD group was increased by 5.34% compared with that in the TD group (Fig. 8b). In addition, the secretory function of the broiler pancreas in the TFRD group was also restored. The levels of LIPA (*p* < 0.001) and AMYL (*p* < 0.001) were increased in broilers from the TFRD group compared with the TD group, and the INS level was not altered in the TFRD group (Fig. 8c). Importantly, the GLU levels (*p* < 0.001) and the *GLUT8* (*p* < 0.001) and *GLUT12* mRNA expression levels (*p* < 0.001) were extremely significantly lower in the TFRD group than in the TD group (Fig. 8d).

The morphological observations revealed that the tibia was significantly hypoplastic with a short and thin tibial backbone in the TD group compared with the TFRD group. Moreover, the white cartilage thrombus area was still thickened considerably in the TD group, while no significant difference in the TGP was observed between the TFRD and CON groups (Fig. 8e). The results of the quantitative bone histomorphologic analysis are shown in Fig. 8f-h. Further pathological observations of the tibial epiphysis showed that the TFRD group had more blood vessels in the hypertrophic zone than the TD group, and the calcification was increased compared with the TD group (Fig. 8e).

In terms of blood vessels, small vessels were observed in the tibial hypertrophic zone in the TD group, and the distribution of osteoblasts in the hypertrophic area remained dispersed and showed nuclear consolidation, and nucleolysis in the TD group (Fig. 8i). Conversely, the blood vessels in the hypertrophic zone were more abundant, the osteoblast distribution was relatively dense, and the osteoblast structure was relatively regular in the TFRD group (Fig. 8i). In addition, the mRNA expression levels of *PI3K* (*p* < 0.001), *AKT* (*p* < 0.001) and *VEGFA* (*p* = 0.004) in the growth plate were significantly upregulated in the TFRD group compared with the TD group (Fig. 8j). PI3K (*p* < 0.001), AKT (*p* = 0.006) and VEGFA (*p* < 0.001) protein levels in the growth plate were also significantly upregulated in the TFRD group compared with the TD group (Fig. 8k, l). These results suggested that the reduction in high blood GLU levels inhibits TD lesions by promoting the expression of proteins and mRNAs in the PI3K/AKT/VEGFA signalling pathway.

Furthermore, Spearman’s correlation analysis was performed to investigate the correlations between GLU-related indicators and bone-related parameters and the correlations between the PI3K/AKT/VEGFA signalling pathway and GLU-related indicators and bone-related parameters (Fig. 8m). The dynamic network heatmap showed that GLUT8 and pancreatic GPR109A levels were positively correlated with TGPW (*r* = 0.915 and 0.683, *p* < 0.001 and *p* = 0.042, respectively) and negatively correlated with TMD (*r* = −0.898 and −0.767, *p* < 0.001 and *p* = 0.015, respectively). LIPA and AMYL levels were positively correlated with TL (*r* = 0.900 and 0.983, *p* < 0.001, respectively) and negatively correlated with TGPW (*r* = −0.833 and −0.833, *p* = 0.005 and 0.001, respectively). Undoubtedly, the *PI3K, AKT* and *VEFGA* mRNA levels were significantly negatively correlated with GLU levels (*r* = −0.898, −0.508 and −0.865, *p* < 0.001, = 0.162, and 0.002, respectively), pancreatic GPR109A levels (*r* = −0.763, −0.610 and −0.661, *p* = 0.016, 0.080 and 0.052, respectively) and TGPW (*r* = −0.491, −0.712 and −0.457, *p* = 0.178, 0.031 and 0.215, respectively), while they were significantly positively correlated with LIPA levels (*r* = 0.406, 0.831 and 0.440, *p* = 0.277, 0.005 and 0.235, respectively), tibia lower 1/3 diameter (TLP) (*r* = 0.898, 0.712 and 0.915, *p* < 0.001 =0.031, <0.001, respectively) and tibia weight (TW) (*r* = 0.627, 0.763 and 0.712, *p* = 0.070, 0.016 and 0.031, respectively). Based on these results, PI3K/AKT/VEGFA signalling is closely related to blood GLU levels and gut-related markers in TD broilers.

## Discussion

Dysfunction of the gut microbial community has been linked to several metabolic diseases, such as obesity, nonalcoholic fatty liver, cardiovascular disease, diabetes, osteoporosis and many other diseases^26–29^. In particular, the GM plays an essential role in regulating blood GLU levels^23^. Interestingly, the acceleration of severe bone disease by high GLU levels has been reported in several studies^30–32^. TD is also a common nutritional metabolic leg disease in broilers. In this study, we found that broilers with severe TD generally had elevated blood GLU levels and severe damage to the pancreas. The results revealed pathological stimulation in TD broilers, which resulted in an altered GM composition, increased production of BA metabolites from the gut bacteria *Blautia* and *Coprococcus*, and high levels of the pancreatic butyrate receptor GPR109A, thereby alleviating hyperglycaemia in these animals. The recovery of high GLU levels in TD broilers resulted in increased mRNA and protein expression levels of PI3K, AKT, and VEGFA in blood vessels, leading to the recovery of blood vessel function and distribution on TGP, thereby alleviating the occurrence of TD.

Studies have suggested that GM plays a crucial role in the pathophysiology of obesity, diabetes mellitus type 2 (T2DM), and other metabolic disorders^33,34^. Large metagenome-wide studies have documented that disorders in gut microbial homoeostasis are associated with hyperglycaemia in China and Europe^35,36^. The GM composition also changed substantially with the induction of high GLU levels in RELMβ KO mice^37^ and C57BL/6J mice^26^. However, a study focusing on the effect of GM on GLU metabolism in broilers is still lacking. In the present study, the composition of the GM was significantly different between the TD group and the CON group, and the level of GLU was significantly increased. The results of the FMT experiment showed that with the restoration of GM dysbiosis in TD broilers, the increase in GLU levels was also suppressed. Vrieze et al.^38^ also showed that transferring microbes from nonhyperglycaemic patients into the gut of obese hyperglycaemic patients may improve insulin sensitivity, restore blood GLU levels, and slow obesity. Thus, GM is considered a critical endogenous factor regulating GLU metabolism.

Several studies have reported that the ratio of Firmicutes to Bacteroidetes is increased, leading to elevated GLU levels^22,39^. Firmicutes play an essential role in absorbing dietary calories and fat storage in gut cells, whereas a decrease in Firmicutes abundance may result in a decreased GLU level by reducing their involvement in energy absorption^40^. This study also confirmed that the ratio of Firmicutes to Bacteroidetes in the gut is proportional to the blood GLU levels of broilers.

At the genus level, we analysed the top 20 species in terms of abundance and importance. In this study, two genera were significantly different in the TD group compared with the CON group: *Blautia* and *Coprococcus*. *Blautia* and *Coprococcus* are representative microbiota in patients with high GLU levels, especially *Blautia*^41^, which is significantly associated with the regulation of GLU and lipid homoeostasis^42^. Wang et al.^43^ indicated that the abundance of *Blautia* in the gut was reduced in 14-week-old and high-fat diet-fed mice. In addition, a randomised clinical trial of patients with T2DM and hyperlipidaemia showed that the significant increase in *Blautia* abundance in the GM attenuates the increased blood GLU level^24^. We observed that the plasma GLU level in broilers was positively correlated with the abundances of *Blautia* and *Coprococcus*, in contrast to previous studies^24,43^. This difference may be because previous studies mainly focused on adult animals or patients whose immune systems had matured. However, the induction period in the present study was only 3 days, which indicated acute induction. Chicks aged 0–21 days have a greater stress response, and the immune system is still in the developing stage. These conditions tend to produce negative feedback to regulate blood GLU homoeostasis. Dai et al.^44^ reported that GLU levels were increased in chicken embryos fed l-arginine and that *Blautia* was abundant in the gut. These findings are consistent with the present results.

Gut microbial metabolites, including SCFAs, serve as a link between the microbial community and host homoeostasis that is involved in blood GLU regulation. Thus, SCFAs are proposed to play essential roles in maintaining host energy homoeostasis and insulin sensitivity^29,45^. Both *Blautia* and *Coprococcus* are metabolically enriched butyrate-producing bacteria. BA is essential for maintaining gut health. BA inhibits the growth of harmful pathogens, reduces the intestinal pH, and promotes epithelial cell differentiation, thereby increasing the villus surface area and modulating brush-border membrane (BBM) development and function^46–49^. In summary, these effects positively influence gut morphology and enhance gut mineral bioavailability and absorption. In addition, BA reduces the metabolism of plasma GLU^50^. Cheng et al.^42^ showed an increase in GLU levels in mice but significant decreases in *Coprococcus* abundance and BA levels. Similarly, Upadhyaya et al.^51^ treated 20 patients with metabolic syndrome (MetS) with type 4 resistant starch (RS4), and the results showed that the percentages of fasting GLU and glycosylated haemoglobin levels decreased and the content of BA in faeces increased in the RS4 group. Additionally, a study reported that oral butyrate significantly improved intestinal integrity reduced the F/B ratio and alleviated diabetic symptoms^52^. In the present study, we restored the balance of blood GLU homoeostasis and observed a negative feedback effect on the TD group; intestinal BA levels showed an increasing trend and were positively correlated with *Blautia* and *Coprococcus* abundances. BA is not only an energy source for the host but also binds to its signature receptor GPR109A, inhibits INS signalling and fat accumulation, improves the homoeostasis of GLU and liposomes, regulates the intestinal barrier and inhibits intestinal inflammation and other biological functions^53–55^. Moreover, in GPR109A knockout mice, BA failed to exert its protective effect^56^. Therefore, we measured the GPR109A content in the pancreas to reflect the effect of BA on the inflammation and function of the pancreas. The pancreatic GPR109A level in the TD group was the highest. Thus, BA reduces pancreatic inflammation and improves pancreatic dysfunction by binding to its receptor GPR109A in the pancreas, thereby restoring blood GLU levels. This process may also be one of the mechanisms by which the gut-pancreas axis regulates blood GLU levels.

The imbalance of intestinal homoeostasis and high GLU production increase intestinal membrane permeability and destroy the intestinal barrier^57,58^. The increase in intestinal membrane permeability allows harmful metabolites in the intestine to reach the corresponding target organs through body fluid circulation, causing inflammation and the subsequent development of obesity and diabetes^21,59^. Disruption of the physical intestinal barrier and increased intestinal membrane permeability in high-GLU-treated mice leads to an increased inflammatory environment and damage to the pancreas through the gut-pancreas axis^18^. In the present study, the intestinal tight junction proteins Claudin 1 and Occludin were significantly downregulated in the high-glucose TD broilers, suggesting that the intestinal barrier had been damaged, and the DAO content in plasma had increased, suggesting increased intestinal permeability. Equally important, we also detected large amounts of inflammatory infiltrates in the pancreases of TD broilers with high GLU levels, and the endocrine and exocrine functions of the pancreas were reduced. Thus, our experimental results indicate that the destruction of the intestinal barrier leads to inflammation of the pancreas via the gut-pancreas axis and that the pancreas may act as an intermediate in the GM-mediated regulation of blood GLU levels. However, additional data have shown that the intestinal metabolite lipopolysaccharide acts on the liver through the intestinal barrier to cause endotoxaemia, inflammation and abnormal lipid accumulation, thereby damaging the balance of blood GLU levels^60,61^. Therefore, more in-depth and detailed experiments are required to explore the comprehensive and specific mechanism by which the GM regulates blood GLU levels.

Hyperglycaemic animals are more susceptible to bone disease than nonhyperglycaemic animals^11,12^. Wang et al.^32^ investigated whether hyperglycaemia is one of the risk factors for osteoporosis. High GLU concentrations alter the biomineralization process of osteoblasts, enhance mineralisation, and reduce the mineral content^31^. Insulin is also a signal that regulates the bone formation, which promotes the differentiation of bone marrow-derived stromal cells into osteoblasts by inducing the expression of osteoblast transcription factors^62^. Animal experiments have also confirmed that injecting insulin into rats effectively increases the number of osteoblasts and promotes the formation of strong bones^63^. As shown in the present study, the INS level in the TD group was drastically decreased, the GLU level was dramatically increased, and some tibial indices, such as TW, tibia length (TL), and TMD, were markedly lower in the TD group than those in the CON group. Moreover, transparent white cartilage thrombi without calcification and vascularisation in the growth plate are hallmarks of TD. Our clinical observations and HE staining suggest that the TGP of TD broilers with high GLU levels is the widest, consistent with the conclusion that high GLU levels reduce bone formation.

The differentially expressed tibial genes were mapped to the KEGG database for functional clustering analysis to explore the specific causes of the impaired tibia in TD broiler chickens. The focal adhesion pathway was most clearly differentially expressed. Focal adhesion kinase plays vital roles in vascular morphogenesis and repair^64^ and is involved in vascular endothelial cell motility, proliferation, adhesion and apoptosis via the activation of the PI3K/AKT signalling pathway^65^. HE staining of the tibia revealed significant decreases in the density, number, and distribution of blood vessels in the TD group. The reduction in blood vessels may lead to an insufficient supply of nutrients needed by cells involved in osteogenesis, particularly osteoblasts, osteoclasts, and mesenchymal stem cells^2^. At the same time, the reduction in blood vessels directly leads to the destruction of bone deposits in the calcified area, the termination of calcification, and finally, the formation of white cartilage thrombus^3^. These findings are consistent with our results. In addition, the cartilage cells in the TD group showed nuclear lysis, nuclear pyknosis, and nuclear rupture, which further confirmed the lack of a nutrient supply and the dysplasia of osteocytes due to the reduced blood vessels.

The PI3K/AKT signalling pathway is a classical and critical pathway by which INS regulates blood GLU levels. Activation of insulin receptors will activate downstream GLU metabolism-related proteins such as PI3K/AKT. Abnormal expression of the PI3K/AKT pathway will cause GLU metabolism disorders^66^. A long-term blood GLU disorder will lead to abnormal blood lipid metabolism, which will cause long-term inflammation of blood vessels, rupture of vascular endothelial cells and abnormal PI3K/AKT signalling^67,68^. Hamed et al.^69^ found that vascular endothelial damage in individuals with T2DM is related to the inhibition of the PI3K/AKT pathway. Da et al.^70^ also documented that hyperglycaemia inhibited PI3K/AKT pathway decreased NO production, and induced a predominant vasoconstrictor effect, leading to the formation of vascular endothelial damage. Based on the findings from the present study, the highest GLU levels were detected in the TD group, and the PI3K and AKT mRNA and protein expression levels were profoundly lower than those in the CON group. Additionally, the blood vessels in the TGP of the TD group were significantly reduced. Similarly, in the FMT experiment, *PI3K* and *AKT* mRNA levels were also increased with the attenuation of high blood GLU levels in the FMT-Con group compared with the TD group. These findings are consistent with previous conclusions, suggesting that disturbances in GLU metabolism lead to a decrease in blood vessels through the PI3K/AKT signalling pathway^67–69^.

We measured VEGFA mRNA and protein expression in the vascular-enriched regions of the TGP to further investigate the role of blood vessels in GLU metabolism and bone. VEGFA, a direct inducer of blood vessel formation, acts on the vascular endothelium, stimulates the division and proliferation of vascular endothelial cells, increases the permeability of microvessels, and leads to the formation of blood vessels^71^. VEGFA expression is regulated by the PI3K/AKT signalling pathway, and activation of the PI3K/AKT signalling pathway induces increased transcription of *VEGFA*, thereby promoting increased blood vessel formation^72^. This result was also observed in the present study, where at 21 days, the change in the number of blood vessels was proportional to the VEGFA mRNA and protein expression in the TD, CON and TFRD groups. However, the VEGFA mRNA and protein expression levels in the TD group were higher than those in the CON group on day 7. This phenomenon has also been observed in previous studies, where the VEGFA mRNA and protein expression levels were significantly increased in TD broilers on day 7^2,73^. The potential explanation for this finding is that VEGFA expression was upregulated in a short time after chicks were subjected to acute vascular injury, suggesting that it has proangiogenic activity. Therefore, we concluded that high GLU levels reduce blood vessel formation through the PI3K/AKT/VEGFA signalling pathway, thereby promoting the occurrence of TD in broilers.

TFRD extract is derived from the roots of *Drynaria roosii Nakaike*^74^. It has been indicated to be useful in the treatment and prevention of bone disorders by increasing the proliferation and differentiation of osteoblasts and promoting bone strength^75,76^. In the present study, after 14 days of TFRD treatment, TD symptoms in the TFRD group were significantly reduced, accompanied by the restoration of GM homoeostasis, and the GLU levels and the pancreas almost returned to normal. These results further validated a relationship between GM-mediated GLU homoeostasis through the gut-pancreas axis and TD in chickens. In addition, previous reports also documented that some flavonoids stimulate GLU uptake, inhibit GLU production and lower blood GLU levels^77–80^. Flavonoids are also extensively metabolised by the GM and modulate changes in the GM^81–84^. Our experiments apparently indicate that TFRD treatment restores the dysbiosis of the GM and lowers blood GLU levels in TD broilers. However, the mechanism by which TFRD regulates flavonoid production, flavonoid-producing gene expression, and flavonoid metabolism by the GM and which signalling pathway affects blood GLU levels require further in-depth research.

**Fig. 9:**
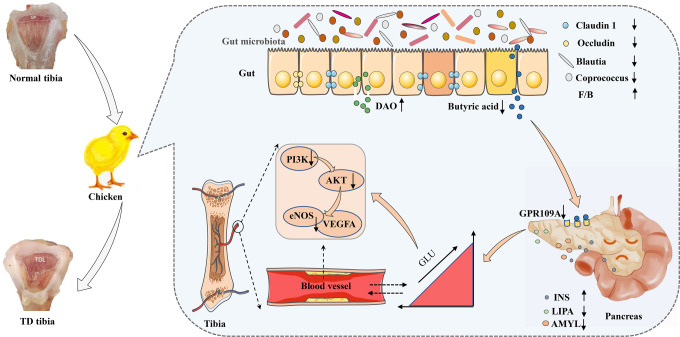
Diagram of the mechanism by which gut microbiome dysregulation drives bone damage in tibial dyschondroplasia by disrupting glucose homoeostasis mediated by the gut–pancreas axis. Impaired gut barrier (reduced expression of Claudin 1 and Occludin) and disordered gut microbiome in broilers leads to reduced abundance of *Blautia*, reduced BA binding to GPR109A receptors in the pancreas, which impairs pancreatic function (decreased LIPA and AMYL levels and increased INS levels) and elevated GLU levels. Elevated GLU levels inhibit the expression of the PI3K/AKT/VEGFA signalling pathway in TGP, thereby exacerbating TD lesions.

In summary, the broiler GM affects the occurrence of TD by regulating GLU metabolism mediated by the gut-pancreas axis (Fig. 9). Specifically, the *Blautia* metabolite BA modulates GLU homoeostasis by acting on the GPR109A receptor in the pancreas. Disturbed GLU metabolism inhibited the expression of the PI3K/AKT/VEGFA signalling pathway in the TGP, thereby exacerbating TD lesions. The pathogenesis of bone diseases is diverse and complex, and our results suggest that GM-mediated GLU metabolism may be a new target to solve bone metabolism-related diseases.

## Methods

### Chemicals, animals and animal ethics

Thiram (AR, purity≥ 98%, #C10036460) was acquired from Macklin Biochemical Co., Ltd. (Shanghai, China). TFRD (purity≥ 95%, #K20798) was purchased from Xi’an Kailai Biological Engineering Co., Ltd. (Xi’an, China). Primary antibodies against Occludin (#WL01996, 1:1000), Claudin 1 (#WL03073, 1:1500), PI3K (#WL02240, 1:1000), and β-actin (#WL01372, 1:1000) and a secondary antibody (HRP-conjugated goat anti-rabbit IgG, #WLA023a, 1:5000 dilution) were purchased from Wanlei Biotechnology Co., Ltd. (Shenyang, China). Primary antibodies against AKT (#GB111114, 1:1000) and VEGFA (#GB111971, 1:1000) were purchased from Servicebio Technology Co., Ltd. (Wuhan, China). Arbour Acres (AA) broiler chickens (male, 1-day-old; 46.96 ± 6.53 g) were acquired from Xingda Poultry Industry Co., Ltd. (Kaifeng, China). The Henan Agricultural University Experimental Animal Ethics Committee approved all animal procedures (Approval No. 17-0126).

### Experimental procedure

All broilers were raised in facilities with a light/dark cycle, standard room temperature (23–35 °C) and relative humidity (60–70%), and were provided access to water and food. All broilers were allowed to adapt to the environment before further experiments, and 120 broilers were randomly divided into CON (*n* = 40) and TD (*n* = 80) groups. A normal diet was provided to broilers in the CON group. The TD group was provided with the same feed supplemented with thiram (100 mg/kg body weight from days 4 to 7). On day 8, the TD model was established, and then the TD group was divided randomly into the TD and TFRD groups. The TFRD group was administered TFRD (500 mg/kg body weight from day 8 to 21). All experiments lasted 21 days.

### Faecal microbiota transplantation

The FMT model was started on day 7, and TD broilers were transplanted with faecal microbiota extracted from CON broilers by gavage (FMT-Con group). The faecal microbiota was extracted from TD broilers and transplanted into CON broilers by gavage (FMT-TD group). Afterwards, the 60 broilers were divided into four groups (*n* = 15/group): the CON, TD, FMT-TD and FMT-Con groups. After collecting fresh faeces, pellets were placed in sterile, precooled PBS containing 10% glycerol. Faeces collected in a single sample were filtered using a stainless-steel mesh (0.25 mm) and stored at −80 °C until further analysis. Receptor chickens were administered 3 mL of the faecal microbiota solution every day for 7 days by gavage.

### Sample collection

Ten chicks from each group were euthanized on day 7 and day 21. Blood was collected aseptically from the wing vein, and the plasma was centrifuged at 4 °C for 10 min at a speed of 3000 × *g* and stored at −20 °C. The pancreas, duodenum, caecal contents and tibia were removed from 10 chickens in each group and stored at −80 °C until further experiments. Samples of the pancreas and tibia were weighed on an electronic balance with a sensitivity of 0.001 g. The TL, TMD, TUP and TLP were measured using Vernier callipers (#SATA91511, TATA Company, Shanghai, China). The proximal tibia was then cut longitudinally in the sagittal plane to reveal the TGP, and the TGPW was measured with Vernier callipers.

### Histopathological analysis

Samples of the pancreas, duodenum and tibia from the experimental groups were fixed with 4% paraformaldehyde. The tibial specimens were decalcified in a decalcifying solution for 48 h before embedding. Then, all specimens were dewaxed with xylene, dehydrated in graded ethanol solutions and embedded in paraffin using a fully enclosed tissue processor (ASP300S, Leica Biosystems, Buffalo Grove, IL, USA). Pancreatic sections (3 μm), duodenal sections (4 μm) and tibial sections (5 μm) were then stained with HE to determine pathological changes.

Histopathological scoring of the duodenum was performed according to three indicators: villus height, crypt depth, and intestinal wall thickness. Each of the three criteria was scored from 0 to 3 points, and the sum of the scores was calculated. The rules for scoring are described in detail in the Supplementary file (Supplementary Table 1).

### Biochemical analysis

The plasma GLU concentration and the pancreatic activities of LIPA and AMYL were detected according to the manufacturer’s instructions (Nanjing Jiancheng Bioengineering Institute, China). Enzyme-linked immunosorbent assays (ELISAs) were used to determine plasma INS, DAO, and GPR109A levels and pancreatic GPR109A expression (Shanghai Mlbio Biotechnology Co., Ltd.).

### RNA extraction and RT-qPCR

Total mRNA was extracted from the broiler pancreases and TGPs using TRIzol reagent and liquid nitrogen. The mRNA quality was measured using a Nanodrop spectrophotometer, and cDNAs were subsequently synthesised. After synthesising cDNAs, 2 SYBR Green I PCR Master Mix (Vazyme Biotech Co., Ltd., China) was used to perform real-time PCR (qPCR). The 2^−ΔΔCT^ calculation method was used to determine the mRNA expression levels. Primers specific for the *GAPDH* mRNA and the target gene sequences, including *GLUT8, GLUT12, AKT, PI3K* and *VEGFA*, were designed by Primerbank and synthesised by Tsingke Biological Technology Co., Ltd. (Beijing, China). The primers utilised in this study are listed in Supplementary Table 2. *GAPDH* mRNA levels were used as the internal control. Gene expression levels were normalised to *GAPDH* expression.

### Western blotting

The duodenum and TGPs from all the groups were initially homogenised in RIPA buffer (#CW2333S, CWBIO, Jiangsu, China). Afterwards, the homogenised mixture was centrifuged for 5 min at 12,000 × *g* to harvest total protein. The total protein content was determined using a BCA protein assay kit according to the manufacturer’s protocol. Equal amounts of proteins (40 μg) were electrophoresed on 4–20% (w/v) SDS-PAGE gels and then transferred to PVDF membranes. Next, the membranes were probed overnight at 4 °C with primary antibodies against Occludin, Claudin 1, AKT, PI3K, VEGFA and β-actin. Then, the membranes were washed with TBST for 30 min, and the secondary antibody was applied and incubated for 2 h at room temperature. The membranes were again washed with TBST for 30 min, and images were captured with an imaging system (AI600, CE, United States). All blots were derived from the same experiment and processed in parallel (Source Data). Finally, the grey level of the exposed strip was analysed using ImageJ software (v1.8.0, NIH).

### 16S rRNA gene sequence analysis

A QIAamp DNA tool mini kit (Qiagen, Hilden, Germany) was used to extract microbial genomic DNA from the caecal contents according to the manufacturer’s instructions. The V3 and V4 regions of 16 S rRNA genes were amplified using the bacterial primers 338 (forwards: 5′-ACTCCTACGGGAGGCAGCA-3′) and 886 (reverse: 5′-GGACTACHVGGGTWTCTAAT-3′). The amplified DNA was extracted from a 2% agarose gel, purified using a QIAquick PCR Purification Kit (Qiagen), and quantified using an Invitrogen dsDNA assay kit (Carlsbad, CA, USA). Then, DNA libraries were constructed, and sequencing was performed on the Illumina MiSeq 250 platform. Raw Illumina read data for all samples were deposited in the NCBI Sequence Read Archive with the accession code PRJNA830845. The 16S sequencing-related data were analysed using Gene Cloud tools (https://www.genescloud.cn).

### SCFA analysis

Samples of the caecal contents were immediately flash-frozen to prevent the loss of SCFAs. A 20 mg sample of the caecal content was mixed with 1 mL of a phosphoric acid solution (0.5% v/v), homogenised, and ultrasonicated for 5 minutes on ice for each sample. The specific methods for analysing SCFAs are described in Supplementary Method 1. To evaluate the SCFAs, the peak areas of AA, PA, BA, isobutyric acid (IBA), isovaleric acid (IVA), and valeric acid (VA) were measured and normalised to 2-methyl valeric acid levels.

### RNA isolation and RNA-seq library preparation

Total RNA was extracted from tibia samples (3–5 mm) using TRIzol reagent. The quality of the mRNA was assessed by Thermo Fisher Scientific, 5225 Verona Road, Madison, USA. RNA-seq library preparation is described in Supplementary Method 2. The structure and expression of differentially expressed genes were analysed, and their functions were annotated based on a comparison with the reference genome.

### Statistical analysis

Data were analysed with SPSS (version 21.0, IBM, USA) and GraphPad Prism (version 8.0.2, San Diego, CA, USA) statistical analysis software. For the GM diversity analysis, Mann‒Whitney U tests and Kruskal‒Wallis one-way ANOVA with LSD post hoc tests were used. Two-tailed Student’s *t* tests and one-way ANOVA with LSD post hoc tests were used to analyse blood GLU- and bone-related indicators, respectively. Heatmaps were constructed by performing Spearman’s correlation analysis using the https://www.omicshare.com/tools/Home/Soft/getsoft website. Spearman’s correlation coefficient was estimated based on the degree of linear correlation of changing trends. The data are reported as means ± SD. Statistical significance was determined as a *p* value of 0.05.

## Supplementary information

Supplementary information

Dataset 1

## Data Availability

The raw 16S rRNA gene sequencing data reported in this paper are available from the National Centre for Biotechnology Information (NCBI) with the Sequence Read Archive (SRA) number PRJNA830845. All other data and the code used to reanalyse the data reported in this paper are available from the corresponding author upon request.

## Code availability

No studies involving custom code or mathematical algorithms were performed.
