# Supplementary material for: Gut microbiome dysregulation drives bone damage in broiler tibial dyschondroplasia by disrupting glucose homeostasis
Source: NPJ Biofilms Microbiomes. 2023 Jan 3;9:1. doi: 10.1038/s41522-022-00360-6 (PMC9810666; doi:10.1038/s41522-022-00360-6)
Supplement: Supplementary file 1 — Supplementary information [file 41522_2022_360_MOESM1_ESM.pdf]

Gut microbiome dysregulation drives bone damage in broiler tibial dyschondroplasia by disrupting glucose homeostasis

Supplementary Methods

Supplementary Method 1:

Short-chain fatty acid (SCFA) analysis

20 mg of caecal contents samples were accurately weighed and placed in a 2 mL EP tube. 1 mL of phosphoric acid (0.5% v/v) solution and a small steel ballwere added to the EP tube. The mixture was grinded for 10 s, three times, then vortexed for 10 min and ultrasonicated for 5 min. 0.1 mL of supernatant was added to 1.5 mL centrifugal tube after the mixture was centrifuged at 12000 xg/min for 10 min at the temperature of 4°C. 0.5 mL MTBE (containing internal standard) solution was added to the centrifugal tube. The mixture was vortexed for 3 min and ultrasonicated for 5 min. After that, the mixture was centrifugedat 12000 xg/min for 10 min at the temperature of 4°C. The supernatant was collected and used for GC-MS/MS analysis.

Agilent 7890B gas chromatograph coupled to a 7000D mass spectrometer with a DB-FFAP column (30 m length × 0.25 mm i.d. × 0.25 μm film thickness, J&W Scientific, USA) was employed for GC-MS/MS analysis of SCFAs. Helium was used as carrier gas, at a flow rate of 1.2 mL/min. Injection was madein the split mode and the injection volume was 2 μL. The oven temperature was held at 90°C for 1 min, raised to 100°C at a rate of 25°C/min, then raised to 150°C at a rate of 20°C/min, held for 0.6 min, raised to 200°C at a rate of 25°C/min, held for 0.5 min, after running for 3 min. All samples were analyzed in multiple reaction monitoring mode. The injector inlet and transfer line temperature were 200 °C and 230 °C, respectively.

Supplementary Method 2:

RNA-seq library preparation

Enrichment of eukaryotic mRNA was performed using a NEBNext Poly(A) mRNA Magnetic Isolation Module (NEB, E7490), and the rRNA and concentrated prokaryotic mRNA were removed using a MICROBExpress Bacterial mRNA Enrichment Kit (Invitrogen, AM1905). mRNA was used as a template to construct the library. The library quality was assessed using the Agilent Bioanalyzer 2100 system (Agilent Technologies, CA, USA). The qualified library was clustered on an Illumina cBot and finally sequenced using an Illumina HiSeq 2500. Prior to analysis, the low-quality reads and rRNA sequences were removed, and the clean reads were mapped to the chicken genome.

Supplementary Figures

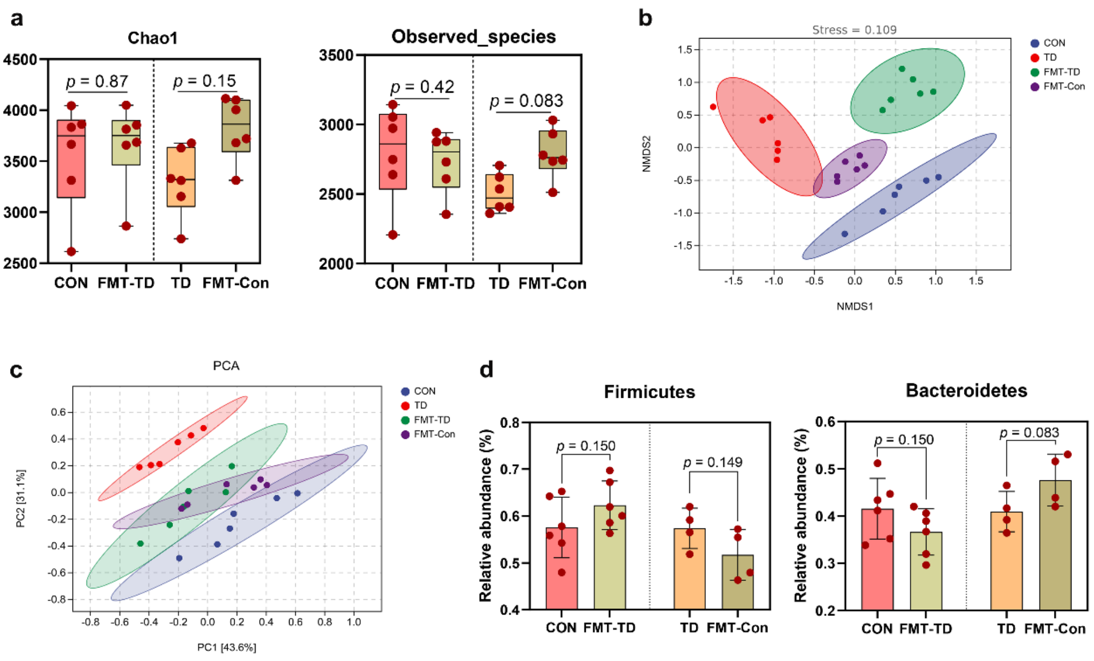

Supplementary Figure1: Gut microbial overall structure in FMT experiment

(a) Chao1 and Observed species indices from FMT experiment were used to analyze the alpha diversity. (b, c) Bray Curits distance-based NMDS and PCA analysis of gut microbiota in CON group (blue), TD group (red), FMT-TD group (green) and FMT-Con group (purple). (d) Differential abundances of Firmicutes and Bacteroidetes at phylum level. n = 6 for each group. A significant difference was defined as a *p value* less than 0.05 calculated using two-tailed unpaired Student's *t* test. The data are presented as the means ± SD.

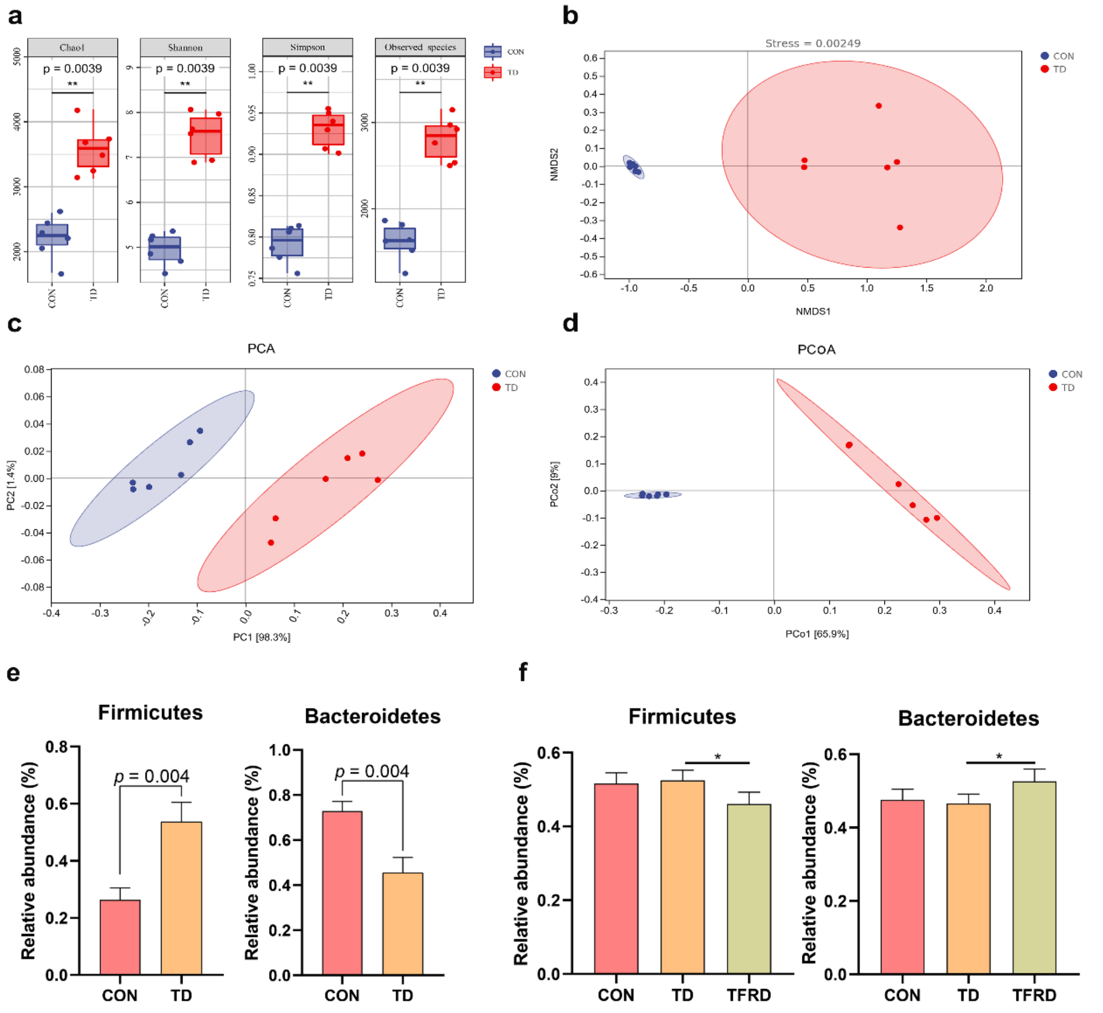

Supplementary Figure2: Gut microbial overall structure in TD and CON broilers

(a) Chao1, Shannon, Simpson and Observed species indices from the CON and TD groups were used to analyze the alpha diversity. (b-d) Bray Curits distance-based NMDS, PCA and PCoA analysis of gut microbiota in the CON group (blue) and TD group (red) of broiler chickens. (e, f) Differential abundances of Firmicutes and Bacteroidetes at phylum level. n = 6 for each group. A significant difference was defined as a *p value* less than 0.05 calculated using two-tailed unpaired Student's *t* test. * $p < 0.05$. The data are presented as the means ± SD.

Supplementary Tables

Supplementary Table 1:

Histological damage score

Score	Villus height (μm)	Crypt depth (μm)	Intestinal wall thickness (μm)
1	200-300	0-30	Over 80
2	100-200	30-40	70-80
3	0-100	40-50	Below 70

Supplementary Table 2:

Primer sequences for quantitative real-time PCR analysis

Gens	Gene bank ID	Primer sequence (5’-3’)	Products length
AKT	NM_205055.1	F: GGCACATTCATTGGCTACAA	107
		R: GGTCGTTCTGTCTTCATCAGC	
PI3K	NM_001004410.1	F: TCGCCACAACAGTAACATCA	120
		R: ACAAAGGGCACACGCTCT	
VEGFA	NM_205042.3	F: CGATGAGGGCCTAGAATGTGTC	101
		R: AGCTCATGTGCGCTATGTGC	
GAPDH	NM-204305.1	F: CCTCTCTGGCAAAGTCCAAG	176
		R: GGTCACGCTCCTGGAAGATA	

F: forward; R: reverse.
