# Supplementary material for: Gut microbiome dysregulation drives bone damage in broiler tibial dyschondroplasia by disrupting glucose homeostasis
Source: NPJ Biofilms Microbiomes. 2023 Jan 3;9:1. doi: 10.1038/s41522-022-00360-6 (PMC9810666; doi:10.1038/s41522-022-00360-6)

Gut microbiome dysregulation drives bone damage in broiler tibial dyschondroplasia by disrupting glucose homeostasis

Results of Western blotting in the duodenum:

Claudin 1 21KDa

Day7 Day21

Group: CON TD CON TD TFRD

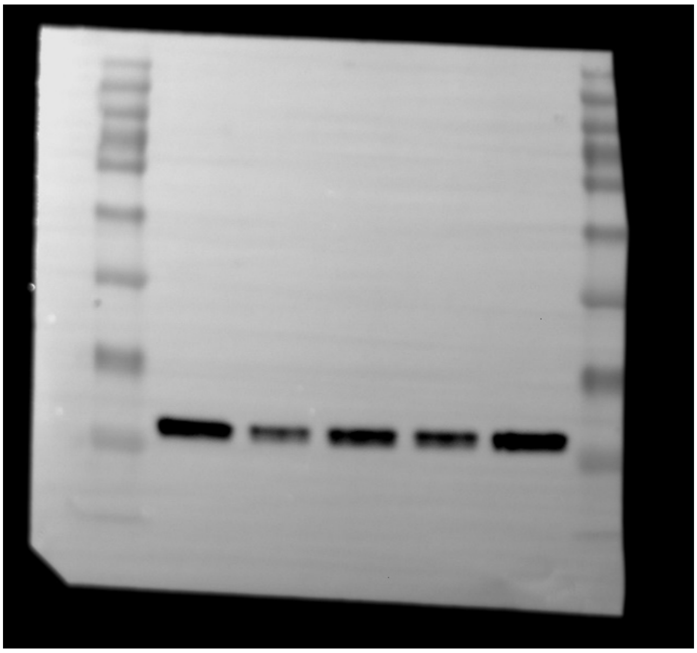

Occludin 60KDa

Day 7 Day 21

Group: CON TD CON TD TFRD

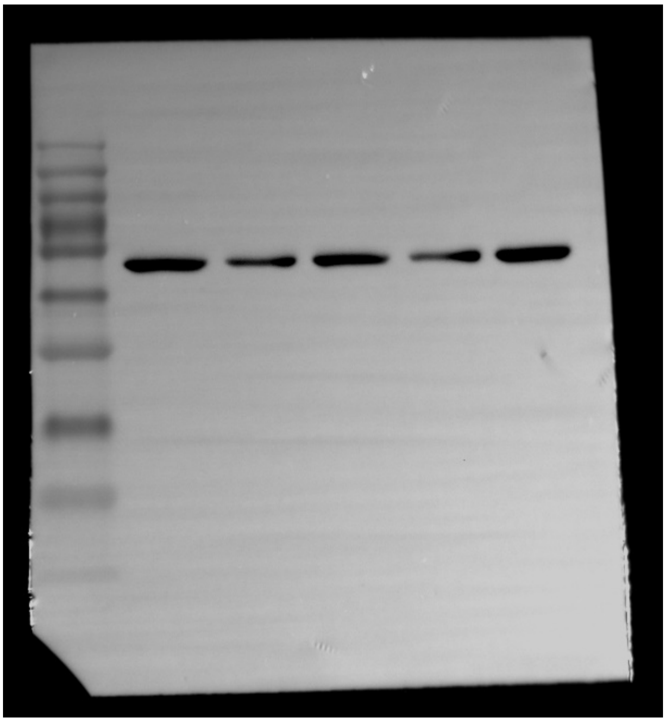

β-actin 43KDa

Day 7 Day 21

Group: CON TD CON TD TFRD

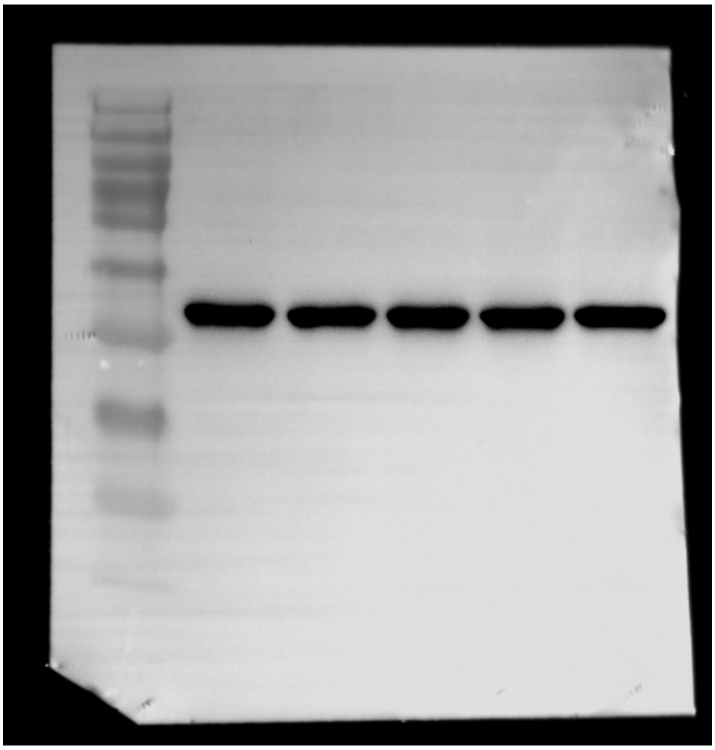

Results of Western blotting in the tibial growth plate:

PI3K 95KDa

Day 7 Day 21

Group: CON TD CON TD TFRD

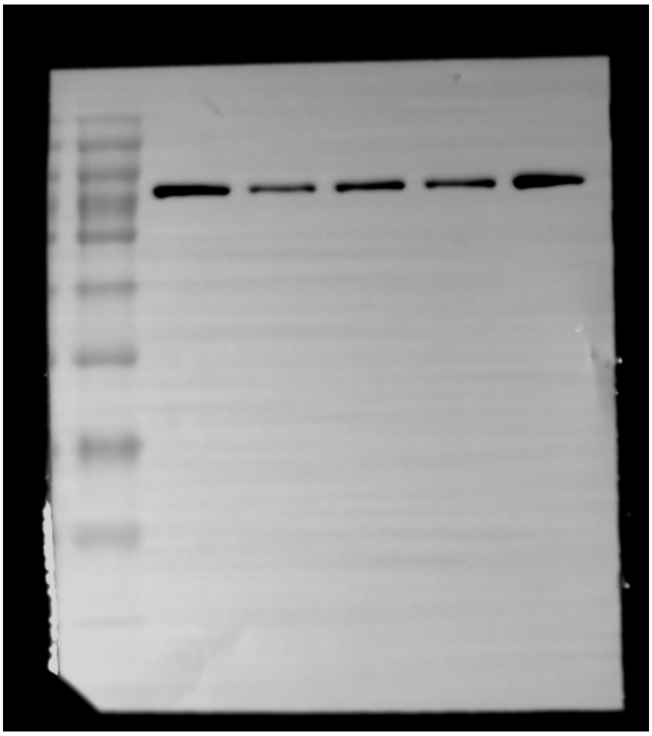

AKT 60KDa

Day 7 Day 21

Group: CON TD CON TD TFRD

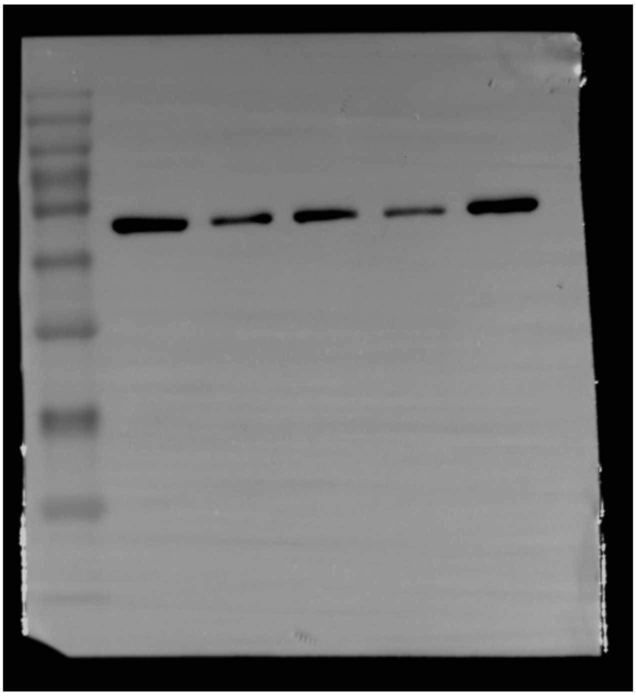

VEGFA 43KDa

Day 7 Day 21

Group: CON TD CON TD TFRD

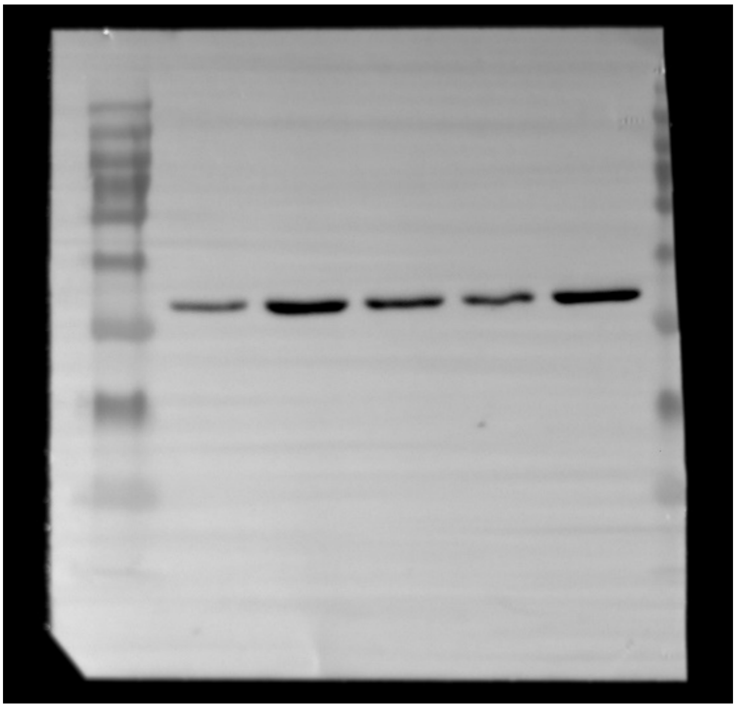

β-actin 43KDa

Day 7 Day 21

Group: CON TD CON TD TFRD

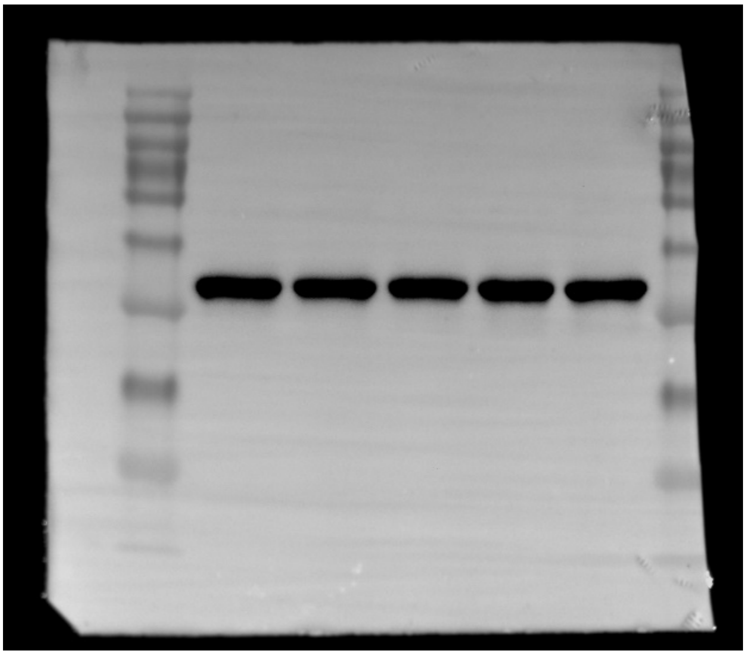

Supplement: Supplementary file 2 — Dataset 1 [file 41522_2022_360_MOESM2_ESM.pdf]
